# Mortality trends and disparities in older U.S. adults with atrial fibrillation and COPD: a 1999–2020 CDC WONDER analysis with forecast to 2030

**DOI:** 10.3389/fcvm.2026.1811188

**Published:** 2026-06-23

**Authors:** Xiao-Bin Zheng, Bing-Qi Yao, Ming Zhang, Shu-Xian Hou, Hai-Yan Wu

**Affiliations:** Department of Cardiology, Shanxi Key Laboratory of Heart Failure Precision Medicine, Shanxi Cardiovascular Hospital, Taiyuan, China

**Keywords:** ARIMA model, atrial fibrillation, CDC WONDER database, chronic obstructive pulmonary disease, mortality trends, older adults

## Abstract

**Background:**

Atrial fibrillation (AF) is prevalent in elderly individuals with comorbidities, particularly those with chronic obstructive pulmonary disease (COPD), where their coexistence increases mortality risk. This study examines mortality trends and disparities among U.S. adults aged 65 and older affected by both AF and COPD from 1999 to 2020.

**Methods:**

We analyzed CDC WONDER mortality records for deaths with both AF and COPD listed on the death certificate. Age-adjusted mortality rates (AAMRs) were assessed, and temporal trends were evaluated using annual and average annual percent changes (APC/AAPC) via Joinpoint regression. An ARIMA model was used to project mortality rates through 2030.

**Results:**

A total of 399,413 deaths were recorded. The AAMR increased from 27.39 in 1999 to 65.29 in 2020, reflecting an AAPC of 4.21 (95% CI: 3.75–4.68). Men had a higher AAMR (57.01) than women (34.96). Non-Hispanic White individuals had an AAMR 2.8 times higher than Hispanics. Non-metropolitan areas had a higher AAMR than metropolitan areas. The leading causes of death were COPD, ischemic heart disease, and cancer, with the most common place of death shifting from medical facilities to home by 2020. Projections suggest the AAMR will reach 92.62 by 2030, with the highest increase observed in individuals aged ≥85.

**Conclusion:**

Mortality involving coexisting AF and COPD in older adults has increased over the past two decades, with disparities across sex, race, and geography. Projections indicate continued mortality increases, emphasizing the need for targeted interventions in high-risk populations.

## 1 Introduction

Atrial fibrillation (AF), a form of irregular heartbeat, is the most prevalent sustained cardiac rhythm disorder and represents a leading cause of stroke, heart failure, and premature deaths globally. According to the analysis of the burden of disease in the year 2021, a total of 52.6 million people worldwide suffered from AF or atrial flutter in 2021, with millions affected annually (1). AF prevalence is known to progressively rise with age. Based on data collected in the Framingham Heart Study, approximately one in four adults aged 40 years or older face a lifetime risk of AF (2). More recent studies demonstrate that this risk is approximately one in three during mid-life among those with one elevated risk factor, including those that are overweight, making way for the cumulative risk of cardiometabolic risk factors in the aging population (3, 4). Chronic obstructive pulmonary disease (COPD) is likewise highly prevalent among adults, with an estimated 213 million individuals affected globally in 2021 (5). Although age-standardized mortality has decreased, the numbers of cases and deaths continue to increase as populations grow and age. Older men with prolonged tobacco exposure experience a heavier COPD burden and are often accompanied by cardiovascular comorbidities, which together contribute to greater mortality in later life (5).

Beyond aging and tobacco exposure, COPD and AF interact through several biological mechanisms. In COPD, chronic hypoxemia, systemic inflammation, oxidative stress, autonomic imbalance, pulmonary hypertension, and hyperinflation-related atrial stretch may promote atrial remodeling and facilitate AF initiation and persistence (6–8). COPD treatments such as β_2_ agonists may modify arrhythmic risk, while cardiovascular comorbidity and endothelial dysfunction further increase susceptibility (6–8). At the population level, COPD is a common comorbidity in patients with AF. In a meta-analysis of 46 studies including 4.2 million patients with AF, COPD was present in 13% of patients, with higher estimates in North American cohorts. Concomitant COPD was associated with greater comorbidity burden and higher risks of all-cause death, cardiovascular death, and major bleeding (9). Contemporary registries confirm management challenges in COPD with AF, showing lower use of beta blockers, variable treatment patterns, and higher risks of death, major cardiovascular events, and major bleeding, while thromboembolic differences are less consistent across regions (10). In COPD cohorts, AF frequently occurs during acute exacerbations and signals worse short-term outcomes. A national Spanish study from 2016 to 2021 found AF in about 20% of hospitalized exacerbations, with higher in-hospital mortality even after adjustment. Older age, male sex, multimorbidity, ventilatory support, and intensive care admission further increased risk (11). In advanced COPD, AF prevalence continues to rise and remains linked to in-hospital death, stroke, and need for mechanical ventilation (12).

Therefore, AF and COPD in later life should not be seen as a simple overlap of two age-related diseases. Their coexistence often reflects multimorbidity, frailty, and competing cardiopulmonary risks. A recent population-based analysis examined mortality among adults aged 25 years and older with AF as the underlying cause of death and COPD as a contributing cause (13). However, that study used a narrower death-certificate definition and included a broader adult population. It did not focus on older adults, a group in whom AF-COPD comorbidity is more common, more clinically complex, and more likely to contribute to fatal outcomes. To address this gap, we used the Centers for Disease Control and Prevention's Wide-Ranging Online Data for Epidemiologic Research (CDC WONDER) database to identify deaths among adults aged 65 years and older with both AF and COPD listed anywhere on the death certificate. This approach aimed to capture a broader measure of mortality involving coexisting AF and COPD. It also reduced heterogeneity from younger patients, who may have different baseline mortality and disease trajectories. This age-specific focus is relevant to geriatric care planning, particularly for decisions about anticoagulation and end-of-life care. We then examined national mortality trends and projected rates through 2030 to inform high-risk population screening and integrated geriatric management strategies.

## 2 Methods

### 2.1 Study design

We queried CDC WONDER Multiple Cause of Death (1999–2020) and identified decedents aged ≥65 years with both AF (ICD-10 I48) and COPD (J41–J44) listed anywhere on the death certificate (underlying or multiple causes) (14, 15). The study followed the Strengthening the Reporting of Observational Studies in Epidemiology (STROBE) guidelines.

### 2.2 Data abstraction

Mortality data were stratified according to key demographic and geographic characteristics. Stratification factors included age, sex, race or ethnicity, age group, place of death, urban–rural designation, census region, state, and underlying cause. Racial and ethnic groups were defined as Hispanic or Latino, non-Hispanic Asian or Pacific Islander, non-Hispanic American Indian or Alaska Native, non-Hispanic Black or African American, and non-Hispanic White. Death location was grouped into hospitals or medical facilities, private residences, nursing or long-term care institutions, hospice centers, and other settings. Age categories followed 10-year intervals from 65 to ≥85 years. Geographic regions were classified as Northeast, Midwest, South, and West. Urbanicity was defined using the National Center for Health Statistics Urban–Rural Classification Scheme, which distinguishes large metropolitan, small-to-medium metropolitan, and non-metropolitan areas.

### 2.3 Statistical analysis

To examine temporal patterns in mortality with AF and COPD, we calculated crude mortality rates and age-adjusted mortality rates (AAMRs) per 100,000 population. AAMRs were standardized to the 2000 U.S. standard population using direct standardization, whereas crude mortality rates were used for age-stratified analyses and were calculated by dividing deaths with AF and COPD by the corresponding annual population. Rates were stratified by demographic, geographic, and clinical characteristics, and 95% confidence intervals (CIs) were estimated. AAMR rate ratios (RRs) were calculated to compare mortality levels between groups as $RR=AAMR_{\mathrm{group}}/AAMR_{\mathrm{reference}}$. RRs with 95% CIs excluding 1 were considered statistically significant, corresponding to a two-sided *P* < 0.05. Temporal trends in AAMRs were assessed using the Joinpoint Regression Program (version 6.0.1, National Cancer Institute) by fitting weighted log-linear models with year as the independent variable and log(AAMR) as the dependent variable. The standard errors of AAMRs were used to account for heteroscedasticity, and errors were modeled as uncorrelated. The annual percent change (APC) was calculated as 100×[exp(β)−1], where β is the slope from the log-linear model, and the average annual percent change (AAPC) was calculated as $100\times \left[\exp\left(\sum w_{i}\beta_{i}/\sum w_{i}\right)-1\right]$, where $\beta_{i}$ and $w_{i}$ represent the slope and length of segment *i*, respectively. Following Joinpoint methodological guidance, the maximum number of joinpoints was determined according to the number of annual observations. Because this study included 22 annual time points from 1999 through 2020, the maximum number of joinpoints was set to 4. Fewer joinpoints were selected when a more parsimonious model adequately characterized the observed trend. The number and location of joinpoints were selected using the data-driven weighted Bayesian information criterion (WBIC), the preferred model selection approach in newer Joinpoint versions. WBIC provides an adaptive, computationally efficient approach that balances model fit and parsimony. The Grid Search method was applied with settings of 2, 2, and 0, requiring at least two observations from either end of the data series to the nearest joinpoint and at least two observations between adjacent joinpoints, with no additional observations skipped when evaluating candidate joinpoint locations. These settings reduced the likelihood that short-term random fluctuations would be interpreted as unstable trend segments. Segment-specific APCs and overall AAPCs were reported with 95% CIs estimated using the parametric method. For between-group trend comparisons, the Joinpoint pairwise comparison function was used. AAPC differences were used to evaluate whether the average annual rates of change differed between groups, whereas parallelism tests were used to assess whether the overall trend patterns were statistically parallel. When annual AAMRs were missing, models were fitted using available annual AAMRs within the relevant calendar interval. If missing values occurred at the beginning of the study period, the trend interval began with the first available year. For isolated missing years within 1999–2020, the AAPC was estimated from the available annual data points within that interval. For pairwise comparisons, the same calendar years were used for the target and reference groups. State-specific estimated annual percent changes (EAPCs) were calculated from available annual AAMRs using weighted log-linear regression with AAMR standard errors used to account for heteroscedasticity. EAPC was defined as 100 × [exp(β) − 1], where β is the slope from the weighted regression of log(AAMR) on calendar year. Statistical significance for APCs, AAPCs, EAPCs, and AAPC differences was assessed using two-sided tests, with statistical significance set at *P* < 0.05. Estimates with 95% CIs excluding 0 were considered statistically significant. Positive estimates with CIs entirely above 0 indicated significant increases, whereas negative estimates with CIs entirely below 0 indicated significant decreases. For parallelism tests, *P* < 0.05 indicated rejection of the null hypothesis that the between-group trends were parallel. Cells with fewer than 10 deaths were suppressed in accordance with CDC WONDER Data Use Restrictions. Suppressed stratum-specific estimates were treated as missing, were not imputed or back-calculated, and aggregate totals were obtained directly from CDC WONDER aggregate queries rather than by summing suppressed cells.

To project future mortality trends up to 2030, we used an autoregressive integrated moving average (ARIMA) model. The optimal model was selected through Grid Search over candidate (p, d, q) values based primarily on the Bayesian Information Criterion (BIC), with Akaike Information Criterion (AIC) reported for comparison. The model was fitted to training data from 1999 to 2014, and adequacy was assessed using the Ljung-Box test. Model robustness was evaluated through rolling-origin cross-validation during a 6-year validation period (2015–2020). Point forecast accuracy was quantified using root mean square error (RMSE), mean absolute error (MAE), mean absolute percentage error (MAPE), symmetric mean absolute percentage error (sMAPE), and mean error (ME, interpreted as model bias). To assess interval forecast performance, we additionally calculated empirical 95% prediction interval (PI) coverage and the mean PI width, including mean PI width as a percentage of the observed value. Variables analyzed included overall mortality as well as stratification by sex and age group.

## 3 Results

### 3.1 Baseline characteristics

**Figure 1:**
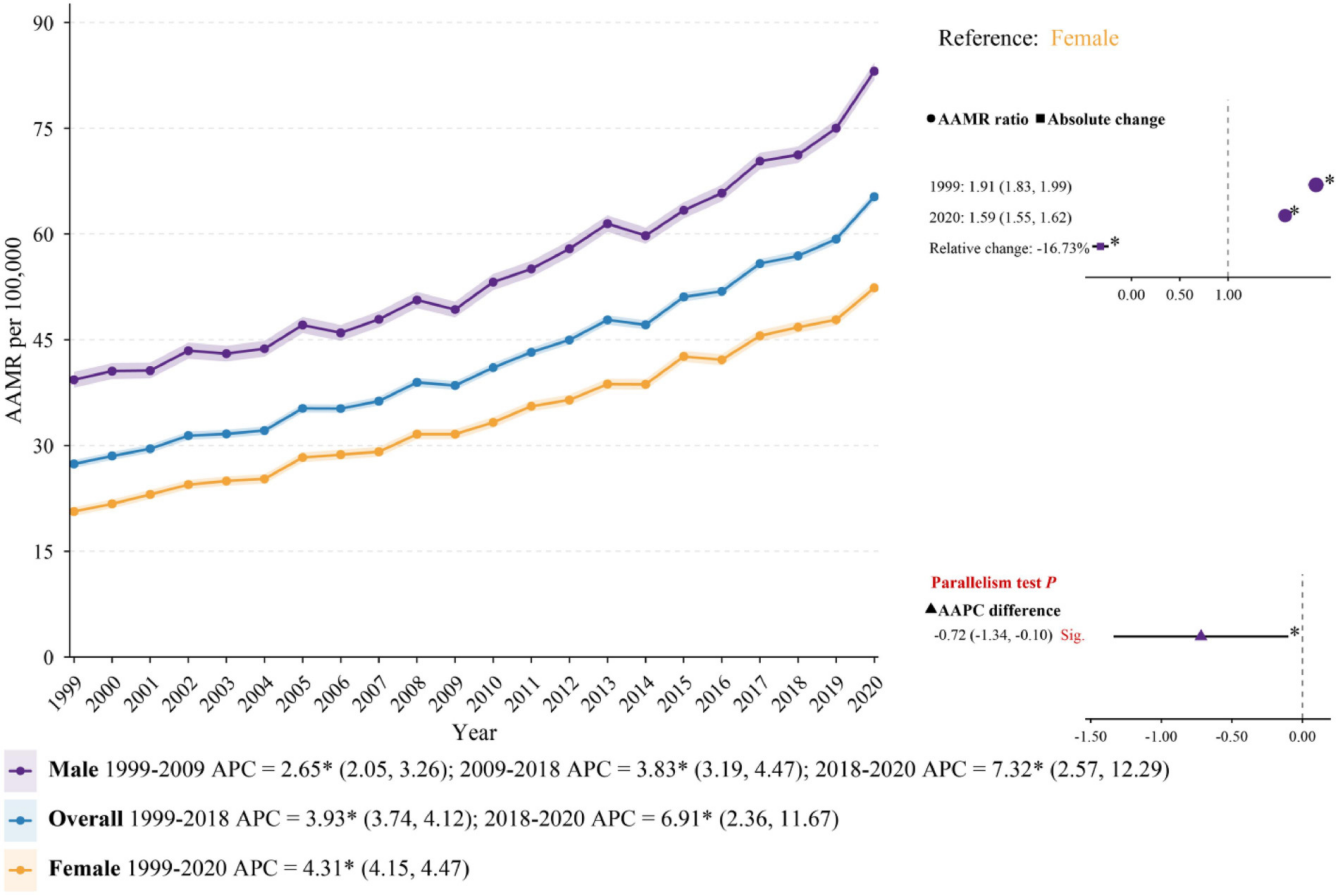
Trends in age-adjusted mortality rates (AAMRs) among older adults with coexisting AF and COPD, stratified by sex in the United States, 1999–2020.

Between 1999 and 2020, 399,413 deaths with AF and COPD were recorded among U.S. adults aged ≥65 years, corresponding to a total population of 928,476,665 and a cumulative crude mortality rate of 43.02 per 100,000. By sex, 204,383 deaths occurred among males and 195,030 among females, with corresponding populations of 400,844,539 and 527,632,126, respectively. The cumulative crude mortality rates were 50.99 per 100,000 among males and 36.96 per 100,000 among females. The overall AAMR over the study period was 43.63 per 100,000 (95% CI: 43.49–43.76), and the AAPC was 4.21 (95% CI: 3.75–4.68). Year-to-year AAMRs rose from 27.39 in 1999 to 65.29 in 2020. Joinpoint analysis identified one inflection point in 2018, with the overall AAMR increasing from 1999 to 2018 (APC = 3.93, 95% CI: 3.74–4.12) and increasing more steeply from 2018 to 2020 (APC = 6.91, 95% CI: 2.36–11.67), indicating a sustained upward trajectory (Table 1, Figure 1, and Supplementary Tables S1, S7).

**Table 1:** Frequency, AAMR per 100,000 and change in older people (≥65 years) with atrial fibrillation and chronic obstructive pulmonary disease concomitantly, 1999–2020.

Variable	Deaths	Population	AAMR (95% CI)	AAPC (95% CI)^a^
Overall	399,413	92,84,76,665	43.63 (43.49–43.76)	4.21 (3.75–4.68)
Sex				
Female	195,030	52,76,32,126	34.96 (34.81–35.12)	4.31 (4.15–4.47)
Male	204,383	40,08,44,539	57.01 (56.77–57.26)	3.59 (3–4.19)
Census region				
Northeast	72,233	17,97,14,300	38.96 (38.68–39.25)	2.83 (2.55–3.12)
Midwest	97,391	20,71,18,111	46.56 (46.27–46.86)	4.35 (4.17–4.54)
South	140,758	34,34,42,253	42.87 (42.65–43.10)	4.69 (4.30–5.09)
West	89,031	19,82,02,001	46.22 (45.91–46.52)	3.83 (3.62–4.04)
Urbanization				
Large metropolitan	174,133	46,83,67,132	37.54 (37.36–37.72)	3.45 (3.26–3.65)
Small-medium metropolitan	138,432	29,21,29,516	48.26 (48–48.51)	4.36 (4.15–4.57)
Non-metropolitan	86,848	16,79,78,547	52.72 (52.36–53.07)	5.16 (4.73–5.60)
Race and ethnicity				
non-Hispanic American Indian or Alaska Native	1,544	60,51,868	30.12 (28.59–31.66)	5.18 (4.34–6.03)
non-Hispanic Asian or Pacific Islander	4,237	35,91,9,615	13.59 (13.18–14)	2.44 (1.75–3.14)
non-Hispanic Black or African American	15,171	83,27,4,492	19.74 (19.42–20.05)	5.41 (4.53–6.30)
non-Hispanic White	378,461	80,32,30,690	47.17 (47.02–47.32)	4.36 (3.88–4.85)
Hispanic or Latino	9,744	65,12,4,722	17.15 (16.81–17.50)	4.57 (3.14–6.02)
Age group^b^				
65–74 years	79,342	51,04,58,341	15.54 (15.44–15.65)	3.88 (3.33–4.44)
75–84 years	164,079	29,85,04,433	54.97 (54.7–55.23)	3.90 (3.43–4.38)
≥85 years	155,992	11,95,13,891	130.52 (129.87–131.17)	4.49 (4.31–4.68)
Top two states by % AAMR Change				
Oklahoma	7,177	11,50,8,359	64.22 (62.74–65.71)	9.58 (8.53–10.63)
South Dakota	1,472	26,94,651	51.53 (48.87–54.19)	6.95 (5.74–8.18)^c^
Bottom two states by % AAMR Change				
Hawaii	1,045	44,86,904	22.75 (21.36–24.14)	1.59 (0.33–2.87)
Alaska	497	12,87,383	47.28 (43.02–51.55)	−3.66 (−7.44–0.27)^d^

AAMR, age-adjusted mortality rate; AAPC, average annual percentage change; CI, confidence interval.

a The AAPC is significantly different from zero at α = 0.05.

b Crude mortality rates were used for age-group analysis.

c South Dakota AAPC was estimated from available 1999–2020 AAMR data excluding 2000 and 2003.

d Alaska AAPC was estimated from available 2003–2020 AAMR data, excluding 2004, 2005, and 2007–2009, and was not statistically significant.

### 3.2 Mortality trends by demographic groups

#### 3.2.1 Sex group

From 1999 to 2020, AAMRs were consistently higher in males than in females. In 1999, the AAMR was 39.34 per 100,000 in males and 20.64 per 100,000 in females. By 2020, these increased to 83.08 and 52.36 per 100,000, respectively (Table 1, and Supplementary Tables S1, S7). The male-to-female AAMR ratio decreased from 1.91 in 1999 to 1.59 in 2020, corresponding to a relative decrease of 16.73%. Joinpoint analysis indicated three phases in males, with an increase from 1999 to 2009 (APC = 2.65, 95% CI: 2.05–3.26), a steeper rise from 2009 to 2018 (APC = 3.83, 95% CI: 3.19–4.47), and further acceleration from 2018 to 2020 (APC = 7.32, 95% CI: 2.57–12.29). Females had a single sustained increase from 1999 to 2020 (APC = 4.31, 95% CI: 4.15–4.47). The parallelism test rejected parallel trends between males and females. The male AAPC was 0.72 percentage points lower than the female AAPC (AAPC difference = −0.72, 95% CI: −1.34–−0.10) (Figure 1).

#### 3.2.2 Age group

Crude mortality rates increased in all age groups, with the greatest burden in the oldest group. In 1999, rates were 10.41, 35.59, and 76.00 per 100,000 for ages 65–74, 75–84, and ≥85 years, respectively. By 2020, they had increased to 23.32, 81.45, and 197.21 per 100,000. Relative to ages 65–74 years, CMR ratios increased from 3.42 to 3.49 for ages 75–84 years and from 7.30 to 8.46 for ages ≥85 years, corresponding to relative increases of 2.12% and 15.79%, respectively (Table 1, and Supplementary Table S2). Joinpoint analysis showed a moderate rise among ages 65–74 years from 1999 to 2007 (APC = 2.19, 95% CI: 1.38–3.01), a stronger increase from 2007 to 2018 (APC = 4.47, 95% CI: 4.03–4.91), and further acceleration from 2018 to 2020 (APC = 7.54, 95% CI: 3.33–11.91). Among ages 75–84 years, rates increased steadily from 1999 to 2018 (APC = 3.65, 95% CI: 3.46–3.84) and accelerated from 2018 to 2020 (APC = 6.33, 95% CI: 1.66–11.22). Among ages ≥85 years, rates rose persistently from 1999 to 2020 (APC = 4.49, 95% CI: 4.31–4.68). Parallelism tests rejected parallel trends for both older age groups compared with ages 65–74 years. The AAPC difference was not statistically significant for ages 75–84 years (AAPC differen = 0.02, 95% CI: −0.71–0.75), whereas ages ≥85 years had a significantly higher AAPC than ages 65–74 years (AAPC difference = 0.61, 95% CI: 0.02–1.19) (Supplementary Figure S1).

#### 3.2.3 Race and ethnicity

**Figure 2:**
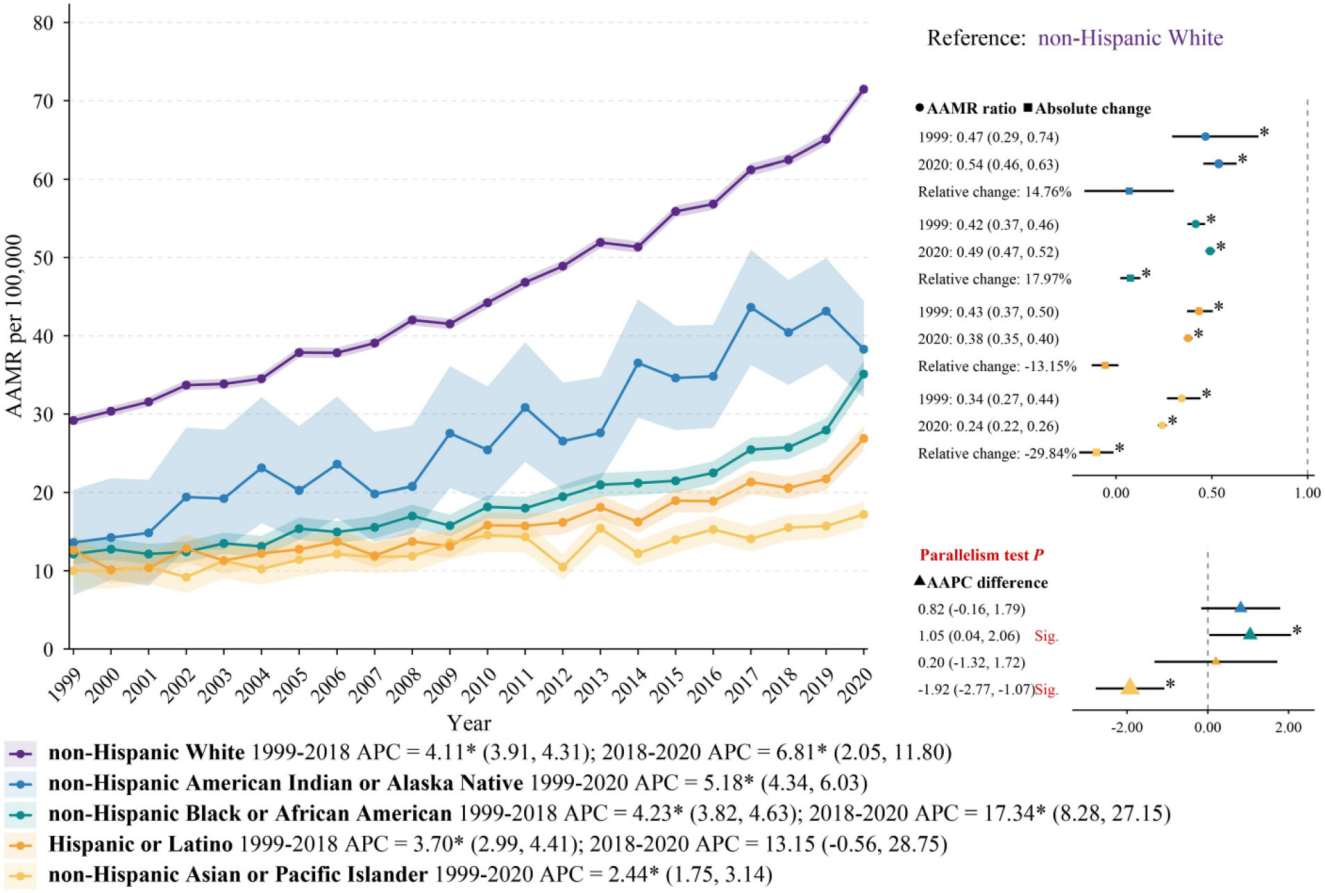
Trends in age-adjusted mortality rates (AAMRs) among older adults with coexisting AF and COPD, stratified by race and ethnicity in the United States, 1999–2020.

From 1999 to 2020, AAMRs increased across all racial and ethnic groups. Over the study period, AAMRs were highest in non-Hispanic White individuals, followed by non-Hispanic American Indian or Alaska Native, non-Hispanic Black or African American, Hispanic or Latino, and non-Hispanic Asian or Pacific Islander individuals. The highest AAPC was observed in non-Hispanic Black or African American individuals (AAPC = 5.41, 95% CI: 4.53–6.30), followed by non-Hispanic American Indian or Alaska Native (AAPC = 5.18, 95% CI: 4.34–6.03), Hispanic or Latino (AAPC = 4.57, 95% CI: 3.14–6.02), non-Hispanic White (AAPC = 4.36, 95% CI: 3.88–4.85), and non-Hispanic Asian or Pacific Islander individuals (AAPC = 2.44, 95% CI: 1.75–3.14) (Table 1, and Supplementary Tables S3, S8). Relative to non-Hispanic White individuals, AAMR ratios remained below 1.00 for all other racial and ethnic groups in both 1999 and 2020. Ratios increased for non-Hispanic American Indian or Alaska Native individuals from 0.47 to 0.54 and for non-Hispanic Black or African American individuals from 0.42 to 0.49, while ratios decreased for Hispanic or Latino individuals from 0.43 to 0.38 and for non-Hispanic Asian or Pacific Islander individuals from 0.34 to 0.24 (Figure 2).

Joinpoint analysis showed heterogeneous upward trajectories across racial and ethnic groups. Non-Hispanic White individuals increased from 1999 to 2018 (APC = 4.11, 95% CI: 3.91–4.31) and accelerated from 2018 to 2020 (APC = 6.81, 95% CI: 2.05–11.80). Non-Hispanic Black or African American individuals increased from 1999 to 2018 (APC = 4.23, 95% CI: 3.82–4.63) and rose sharply from 2018 to 2020 (APC = 17.34, 95% CI: 8.28–27.15). Non-Hispanic American Indian or Alaska Native and non-Hispanic Asian or Pacific Islander groups each followed a single increasing segment, with APCs of 5.18 (95% CI: 4.34–6.03) and 2.44 (95% CI: 1.75–3.14), respectively. Hispanic or Latino individuals increased from 1999 to 2018 (APC = 3.70, 95% CI: 2.99–4.41), followed by a non-significant increase from 2018 to 2020 (APC = 13.15, 95% CI: −0.56 to 28.75). Parallelism tests rejected parallel trends for non-Hispanic Black or African American and non-Hispanic Asian or Pacific Islander groups compared with non-Hispanic White individuals, but not for Hispanic or Latino or non-Hispanic American Indian or Alaska Native groups. AAPC differences were statistically significant for non-Hispanic Black or African American (1.05, 95% CI: 0.04–2.06) and non-Hispanic Asian or Pacific Islander individuals (−1.92, 95% CI: −2.77–−1.07), but not for Hispanic or Latino (0.20, 95% CI: −1.32–1.72) or non-Hispanic American Indian or Alaska Native individuals (0.82, 95% CI: −0.16–1.79) (Figure 2).

### 3.3 Regional and geographical trends

#### 3.3.1 By census region

**Figure 3:**
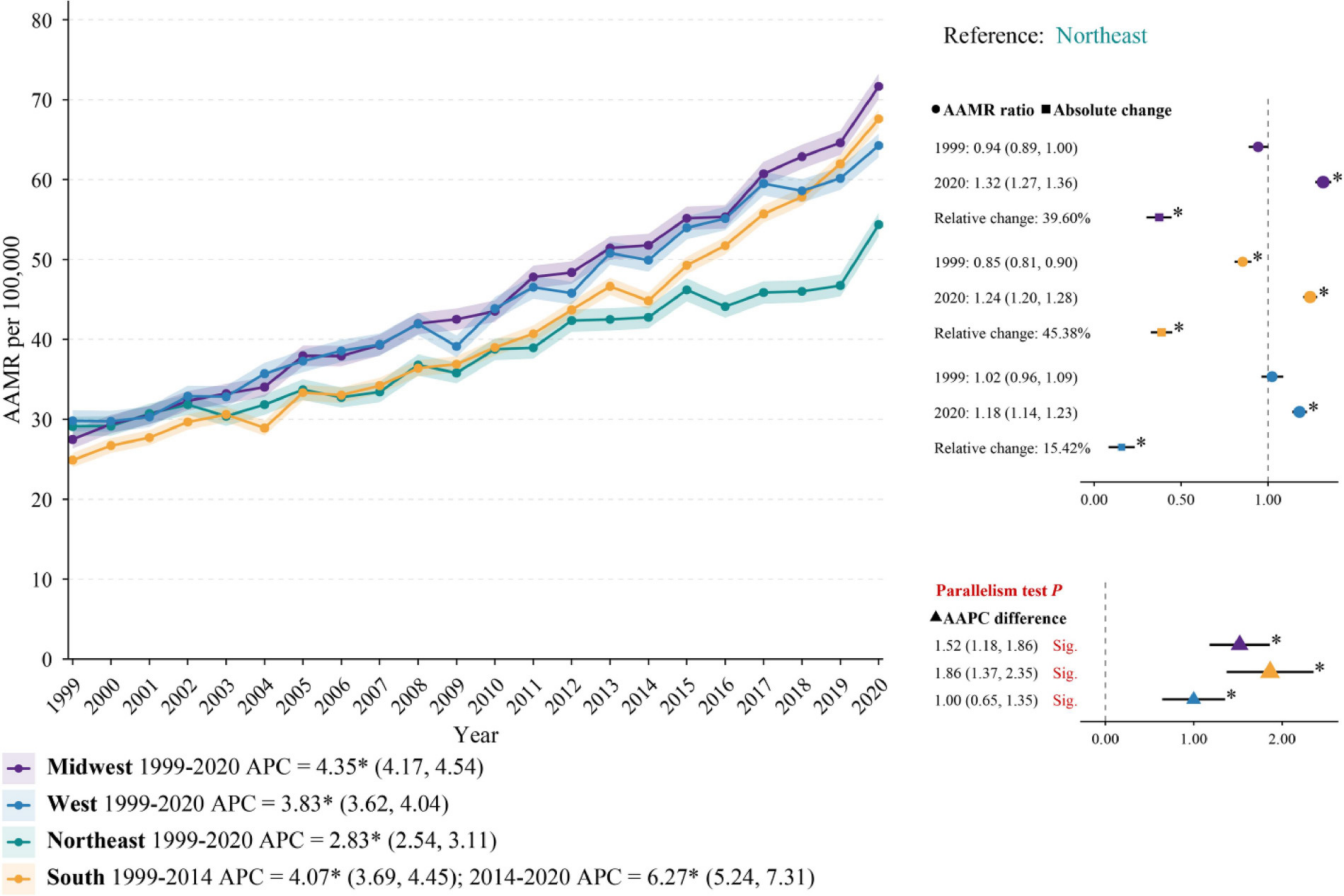
Trends in age-adjusted mortality rates (AAMRs) among older adults with coexisting AF and COPD, stratified by census region in the United States, 1999–2020.

AAMRs increased across all four census regions from 1999 to 2020. Over the full study period, AAMRs were highest in the Midwest (46.56 per 100,000) and West (46.22 per 100,000), followed by the South (42.87 per 100,000) and Northeast (38.96 per 100,000). The AAPC was highest in the South (AAPC = 4.69, 95% CI: 4.30–5.09), followed by the Midwest (AAPC = 4.35, 95% CI: 4.17–4.54), West (AAPC = 3.83, 95% CI: 3.62–4.04), and Northeast (AAPC = 2.83, 95% CI: 2.55–3.12) (Table 1, and Supplementary Tables S4, S9). Using the Northeast as the reference, AAMR ratios increased from 0.94 to 1.32 for the Midwest, from 0.85 to 1.24 for the South, and from 1.02 to 1.18 for the West, corresponding to relative increases of 39.60%, 45.38%, and 15.42%, respectively. Joinpoint analysis showed a single sustained increase in the Midwest, West, and Northeast, with APCs of 4.35 (95% CI: 4.17–4.54), 3.83 (95% CI: 3.62–4.04), and 2.83 (95% CI: 2.54–3.11), respectively. The South increased from 1999 to 2014 (APC = 4.07, 95% CI: 3.69–4.45) and accelerated from 2014 to 2020 (APC = 6.27, 95% CI: 5.24–7.31). Parallelism tests rejected parallel trends for all three regions compared with the Northeast, and AAPC differences were statistically significant for the Midwest (1.52, 95% CI: 1.18–1.86), South (1.86, 95% CI: 1.37–2.35), and West (1.00, 95% CI: 0.65–1.35) (Figure 3).

#### 3.3.2 By urban–rural classification

AAMRs increased across all urbanization categories from 1999 to 2020, with long-run levels highest in non-metropolitan areas (52.72 per 100,000), followed by small-to-medium metropolitan areas (48.26 per 100,000) and large metropolitan areas (37.54 per 100,000). The same gradient was observed for AAPC, with values of 5.16 (95% CI: 4.73–5.60), 4.36 (95% CI: 4.15–4.57), and 3.45 (95% CI: 3.26–3.65), respectively (Table 1, and Supplementary Tables S5, S10). Compared with large metropolitan areas, AAMR ratios increased from 1.11 to 1.36 for small-to-medium metropolitan areas and from 1.16 to 1.63 for non-metropolitan areas, corresponding to relative increases of 21.75% and 40.41%, respectively. Joinpoint analysis showed sustained increases in large metropolitan (APC = 3.45, 95% CI: 3.26–3.65) and small-to-medium metropolitan (APC = 4.36, 95% CI: 4.15–4.57) areas across 1999–2020, while non-metropolitan areas increased from 1999 to 2016 (APC = 4.60, 95% CI: 4.29–4.90) and accelerated from 2016 to 2020 (APC = 7.59, 95% CI: 5.67–9.55). Parallelism tests rejected parallel trends for both groups compared with large metropolitan areas, and AAPC differences were statistically significant (Supplementary Figure S2).

#### 3.3.3 By place of death

Between 1999 and 2020, the distribution of deaths by place of occurrence shifted markedly. In 1999, deaths occurred most often in medical facilities (5,067, 53.8%), followed by nursing/long-term care (2,600, 27.6%), deaths at home (1,560, 16.6%), and other locations (198, 2.1%), while deaths in hospice facilities were 0. By 2020, deaths at home became the most common (11808, 34.6%), closely followed by medical facilities (11,460, 33.6%), with additional deaths in nursing/long-term care (7,014, 20.6%), hospice facilities (2,501, 7.3%), and other locations (1,337, 3.9%). Over the full period, cumulative counts were 163,886 (41.03%) in medical facilities, 101,629 (25.44%) at home, 99,466 (24.90%) in nursing/long-term care, 19,638 (4.92%) in hospice facilities, and 14,794 (3.70%) in other settings (Supplementary Table S6, and Supplementary Figure S3).

#### 3.3.4 By state

**Figure 4:**
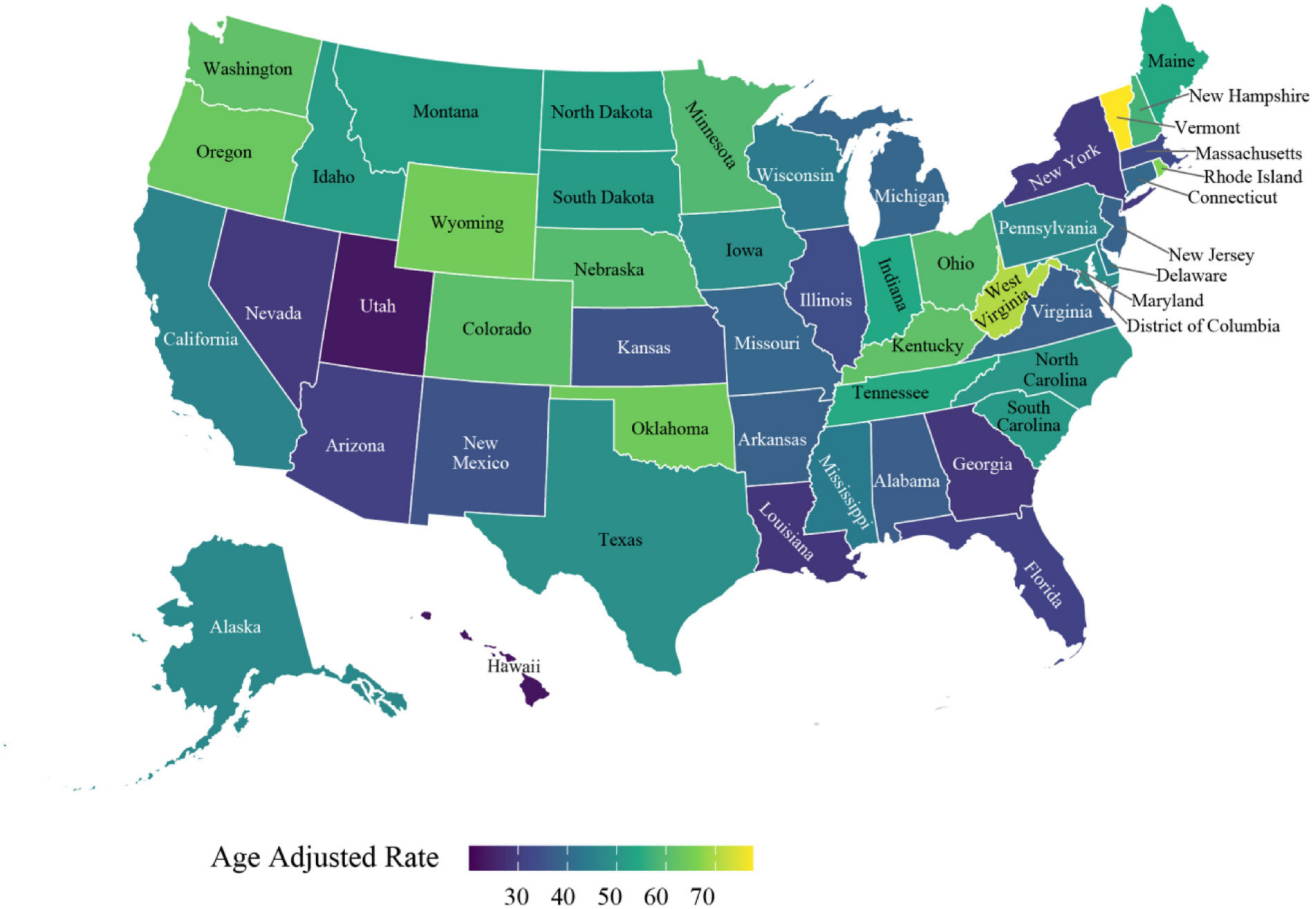
Trends in age-adjusted mortality rates (AAMRs) among older adults with coexisting AF and COPD in the United States, mapped for each state of the United States, 1999–2020.

**Figure 5:**
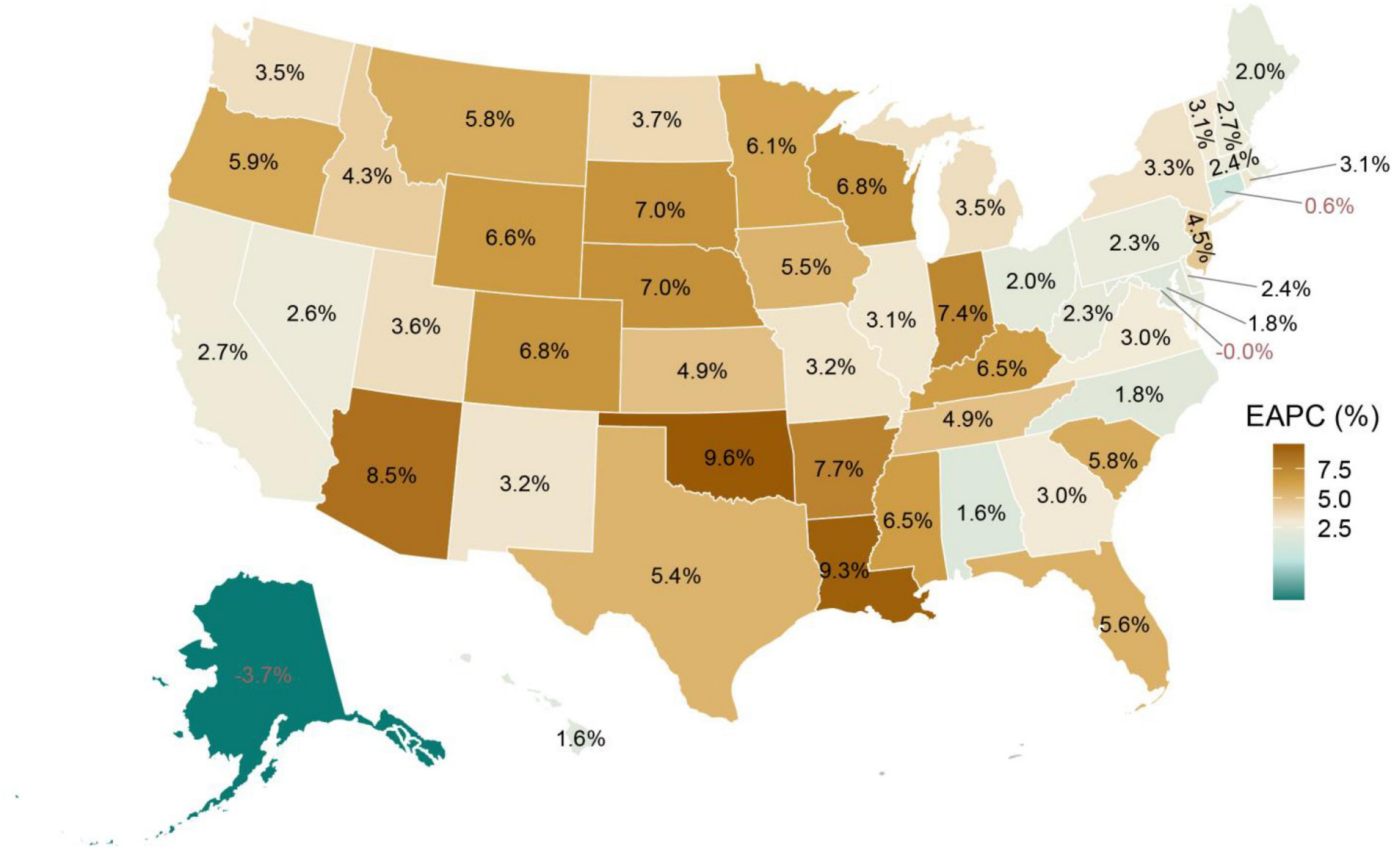
Trends in estimated annual percentage change (EAPC) among older adults with coexisting AF and COPD in the United States, mapped for each state of the United States, 1999–2020. State labels indicate EAPCs and black labels indicate statistical significance.

AAMRs differed substantially across states, ranging from 20.1 per 100,000 in the District of Columbia to 77.6 per 100,000 in Vermont. States in the top 90th percentile included Vermont, West Virginia, Rhode Island, Wyoming, Oklahoma, and Oregon. Their AAMRs were approximately three to four times higher than those in the bottom 10th percentile, which included the District of Columbia, Hawaii, Utah, Louisiana, Georgia, and Nevada (Figure 4, Supplementary Table S12). From 1999 to 2020, the largest percentage increases in AAMR occurred in Oklahoma (759.29%) and South Dakota (496.82%), whereas the smallest changes occurred in Alaska (−25.12%) and Hawaii (32.18%). Parallelism tests rejected parallel trends for Oklahoma, South Dakota, and Alaska compared with Hawaii. AAPC differences were statistically significant for Oklahoma (7.99, 95% CI: 6.34–9.64), South Dakota (5.36, 95% CI: 3.60–7.12), and Alaska (−5.25, 95% CI: −9.31–−1.19) (Table 1, Figure 4, Supplementary Tables S11, S12, and Supplementary Figure S4). Among states with sustained annual increases based on EAPC, Oklahoma had the fastest growth (EAPC = 9.58, 95% CI: 8.53–10.63), while Hawaii had the slowest (EAPC = 1.59, 95% CI: 0.33–2.87) (Figure 5).

### 3.4 Leading underlying causes of death

**Figure 6:**
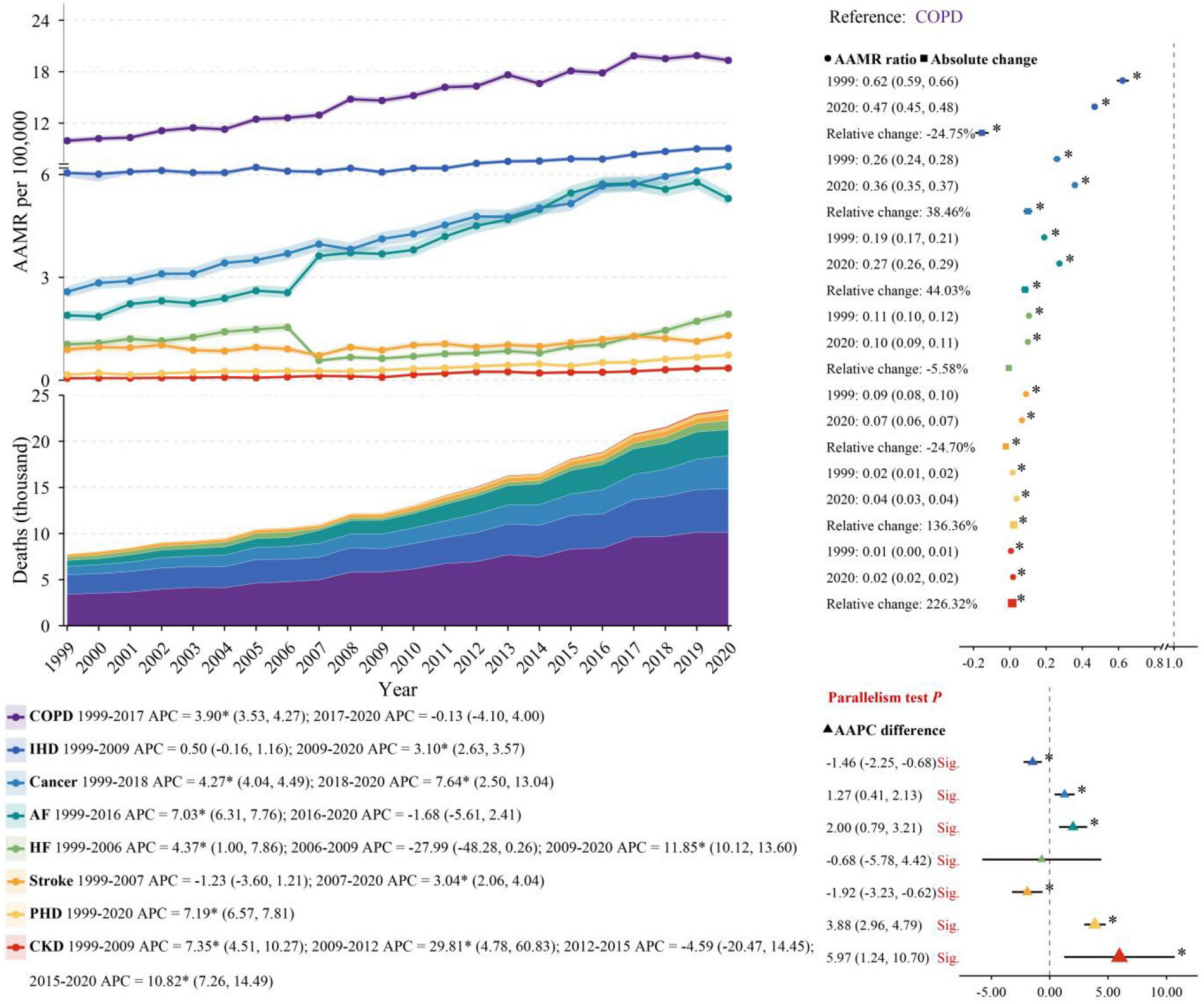
Trends in age-adjusted mortality rates (AAMRs) and number of deaths by several underlying causes among older adults with coexisting AF and COPD in the United States, 1999–2020.

Deaths and AAMRs increased from 1999 to 2020 for most leading underlying causes. COPD had the highest AAMR throughout the study period, increasing from 9.9 to 19.3 per 100,000. Ischemic heart disease (IHD) increased from 6.2 to 9.0 per 100,000, cancer from 2.6 to 6.9 per 100,000, AF from 1.9 to 5.3 per 100,000, heart failure from 1.0 to 1.9 per 100,000, stroke from 0.9 to 1.3 per 100,000, and pulmonary heart disease from 0.2 to 0.7 per 100,000. Chronic kidney disease increased from 0.1 per 100,000 in 2000 to 0.4 per 100,000 in 2020 and remained the lowest category (Supplementary Table S13). Joinpoint analysis showed that COPD, the leading underlying cause, increased from 1999 to 2017 (APC = 3.90, 95% CI: 3.53–4.27), followed by no significant change from 2017 to 2020 (APC = −0.13, 95% CI: −4.10 to 4.00). IHD remained stable from 1999 to 2009 and then increased from 2009 to 2020 (APC = 3.10, 95% CI: 2.63–3.57). Cancer increased from 1999 to 2018 and accelerated during 2018–2020 (APC = 7.64, 95% CI: 2.50–13.04), whereas AF increased through 2016 and then showed no significant change. Heart failure showed the steepest late increase, rising from 2009 to 2020 (APC = 11.85, 95% CI: 10.12–13.60). Pulmonary heart disease and chronic kidney disease had the largest relative increases compared with COPD, despite lower absolute mortality. Parallelism tests rejected parallel trends for all causes compared with COPD. AAPC differences were significant for IHD, cancer, AF, stroke, pulmonary heart disease, and chronic kidney disease, but not for heart failure (Figure 6). Detailed Joinpoint model selection results, pairwise parallelism test results, and WBIC-based model selection are provided in Supplementary Tables S14, S15, and Supplementary Figure S5.

**Figure 7:**
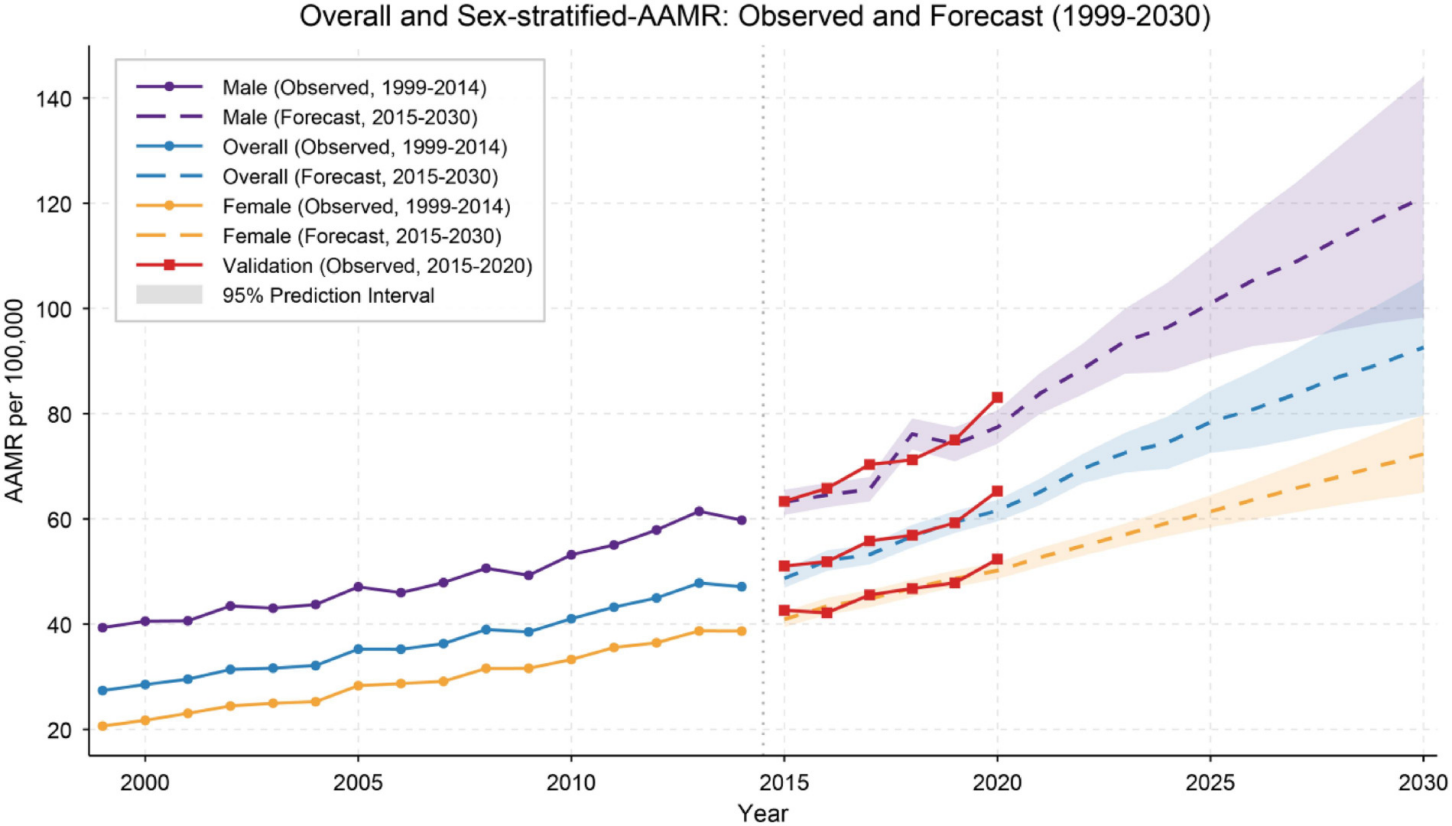
Forecasting age-adjusted mortality rates (AAMRs) among older adults with coexisting AF and COPD, overall and stratified by sex in the United States, 1999–2030.

### 3.5 Projections to 2030

#### 3.5.1 Overall and by sex

Model performance during the 2015–2020 validation period was generally good for the overall and sex-stratified forecasts. RMSE ranged from 1.30 to 3.66, MAE from 1.10 to 2.92, and MAPE from 2.38% to 3.95%. Mean error was negative for all three models, indicating slight underprediction. Empirical 95% prediction interval coverage ranged from 50.0% to 66.7%, suggesting that uncertainty may have been underestimated. However, coverage estimates should be interpreted cautiously because the validation period included only six annual observations. The overall AAMR is projected to rise from 43.63 per 100,000 in 2020 to 92.62 per 100,000 in 2030 (AAPC = 3.84, 95% CI: 3.59–4.09), modeled by ARIMA (2, 2, 1) with a BIC of 50.80. Among females, rates are projected to increase from 34.96 to 72.36 per 100,000 (AAPC = 3.58, 95% CI: 3.47–3.69) under ARIMA (0, 2, 2) (BIC = 43.28). Among males, rates are projected to rise from 57.01 to 121.13 per 100,000 (AAPC = 4.10, 95% CI: 3.87–4.33), modeled by ARIMA (2, 2, 1) (BIC = 59.93) (Table 2, Figure 7, and Supplementary Tables S16–S18).

**Table 2:** Optimal ARIMA model parameters and performance by overall, sex, and age group.

Category	Model (p, d, q)	AIC	BIC	Ljung-Box (*P*)	RMSE	MAE	MAPE (%)	sMAPE (%)	ME (Bias)	95% PI coverage (%)	Mean 95% PI width	Mean 95% PI width (% of actual)
Overall	(2, 2, 1)	48.239	50.795	0.737	2.089	1.536	2.658	2.723	−1.416	50.00	3.926	6.946
Sex												
Female	(0, 2, 2)	41.358	43.276	0.724	1.305	1.104	2.382	2.403	−0.425	66.67	3.158	6.863
Male	(2, 2, 1)	57.369	59.925	0.745	3.658	2.924	3.951	3.995	−1.287	50.00	5.448	7.601
Age^a^												
65–74 years	(2, 2, 0)	13.487	15.404	0.748	0.873	0.729	3.562	3.623	−0.420	33.33	1.402	7.011
75–84 years	(0, 2, 1)	59.634	60.912	0.732	3.106	2.331	3.164	3.242	−1.761	83.33	6.676	9.469
≥85 years	(2, 2, 0)	83.846	85.763	0.693	4.569	4.018	2.381	2.415	−3.153	100.00	14.754	8.637

ARIMA, autoregressive integrated moving average; AIC, Akaike information criterion; BIC, Bayesian information criterion; RMSE, root mean square error; MAE, mean absolute error; MAPE, mean absolute percentage error; sMAPE, symmetric mean absolute percentage error; ME, mean error; PI, prediction interval.

The final selected models and the corresponding AIC, BIC, and Ljung-Box test results were derived from the training period (1999–2014). Other forecast performance metrics were calculated during the 2015–2020 validation period.

a Crude mortality rates were used for age-group analysis.

#### 3.5.2 By age group

**Figure 8:**
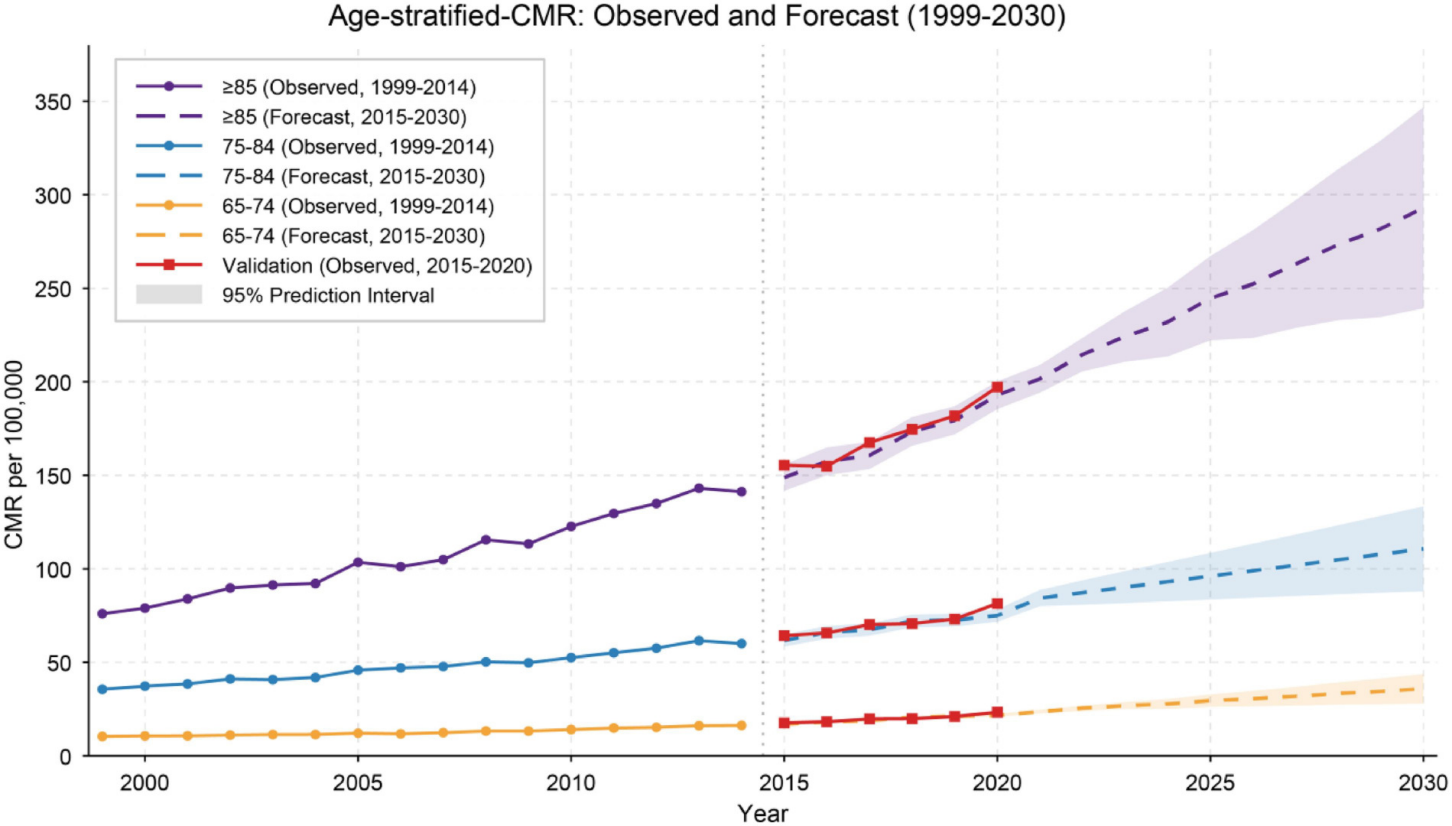
Forecasting crude mortality rates among older adults with coexisting AF and COPD, stratified by ten-year age groups in the United States, 1999–2030.

Across age-stratified models, validation performance was likewise favorable, with RMSE ranging from 0.87 to 4.57, MAE from 0.73 to 4.02, and MAPE from 2.38% to 3.56%. Mean error was consistently negative, again indicating mild underprediction. Prediction interval coverage varied more across age groups, ranging from 33.3% in those aged 65–74 years to 100.0% in those aged 85 years or older, suggesting heterogeneous interval calibration among age groups. For ages 65–74, the crude mortality rate increases from 15.54 to 35.87 per 100,000 (AAPC = 4.59, 95% CI: 4.30–4.88) with ARIMA (2, 2, 0) (BIC = 15.40). For ages 75–84, rates rise from 54.97 to 110.71 per 100,000 (AAPC = 3.06, 95% CI: 2.98–3.14) under ARIMA (0, 2, 1) (BIC = 60.91). For ages ≥85, rates increase from 130.52 to 293.06 per 100,000 (AAPC = 4.13, 95% CI: 3.91–4.34) with ARIMA (2, 2, 0) (BIC = 85.76) (Table 2, Figure 8, and Supplementary Tables S16, S19).

## 4 Discussion

Singh et al. reported 37,738 AF-related deaths with COPD as a contributing cause in adults ≥25 years (1999–2020) using CDC WONDER data (13). In the same database, we identified 399,413 deaths with concurrent AF and COPD in adults ≥65 years. In this national analysis of older adults, mortality involving coexisting COPD and AF increased steadily. Mortality rates were higher in men than in women and rose steeply with advancing age and the greatest burden observed in adults aged 85 years or older. There were marked disparities by race and ethnicity, geographic region, and urban-rural classification. At the same time, the place of death for older adults with coexisting AF and COPD shifted over the study period, with deaths at home and in hospice accounting for a larger share by the end of follow-up. These findings are consistent with the growing burden of AF and COPD and underscore the need to manage cardiopulmonary multimorbidity in older adults. AF incidence and prevalence of disability have increased substantially since 1990, with increasing numbers of COPD cases driven by the aging population and continued exposure to risk factors (1, 5). Current guidelines recommend comprehensive risk factor modification as a cornerstone of management, directly relevant to older populations with co-existing AF and COPD (16, 17). Moreover, recent work emphasises the syndemic nature of COPD and its frequent clustering with other chronic conditions, reinforcing the need to view COPD-AF not just as two diseases but as a constellation of interacting morbidities (18).

### 4.1 Temporal patterns and late-period acceleration

The prior study found that mortality with AF as the underlying cause increased until 2016 and then plateaued, whereas mortality with AF as a contributing cause continued to rise from 2010 to 2020 (19). Singh et al. similarly showed that mortality with AF as the underlying cause and COPD as a contributing cause increased through 2015 and then plateaued from 2015 to 2020 (13). However, after applying a more restrictive definition of COPD and excluding ICD code J40 (bronchitis, not specified as acute or chronic), which had been included by Singh et al. but is not generally considered a specific COPD code, the mortality with AF as the underlying cause in our analysis showed a joinpoint in 2016 followed by a plateau. In parallel, our geriatric multiple-cause approach to coexisting AF and COPD identified a larger mortality burden, with a joinpoint in 2018 and a steeper increase during 2018–2020. The first pandemic year may have contributed to that late acceleration, but the shift should be interpreted cautiously because only 2020 falls within the coronavirus disease 2019 (COVID-19) pandemic period of our dataset. This increase may not be attributable to COVID-19 alone and may also reflect deferred care and disruption of chronic disease management (20). During the first pandemic wave, elective cardiovascular procedures declined by 76%, and outpatient pulmonary care was reduced in many U.S. health systems (21). This is likely to lead to underutilization of rhythm management strategies combined with prevention of crisis in high-risk individuals. Multiple-cause-of-death analyses revealed changes in death certification practices during 2020–2021, potentially influencing attribution of cardiopulmonary comorbidities (22). Regional differences in health-system capacity during this period may have contributed to variation across U.S. states (23, 24).

### 4.2 Age and sex differences

Stratified analyses revealed increased mortality in males and older age groups. The pronounced age-related rise in AF and COPD burden is well documented (1, 5). Smoking history, especially in elderly males, remains a leading COPD risk factor (5), with cumulative exposure heightening vulnerability. COPD exacerbates AF through multiple mechanisms, gaining significance with aging and frailty (6–8).

In the prior study, women had more deaths from AF with COPD as a contributing cause, whereas men had higher AAMRs (13). In our cohort, both deaths and AAMRs were higher in men, but the faster increase among women (AAPC, 4.31 vs. 3.59 in men) suggests a rising female burden of mortality with coexisting AF and COPD, potentially worsened by underdosed anticoagulation in women with AF (25). A large U.S. outpatient cohort analysis showed women had a 10% lower adjusted likelihood of receiving oral anticoagulants than men (26). This treatment disparity, despite women's lower baseline risk, may worsen outcomes. Sex-specific biological and pharmacokinetic differences contribute to this gap. Women tend to have higher plasma concentrations of direct oral anticoagulants (DOACs) due to lower body weight and lower clearance, potentially increasing bleeding risk if standard dosing is used (27). Hormonal factors, notably estrogen's cardioprotective effects, may delay atrial remodeling and inflammation, explaining lower pre-menopausal AF incidence in women (28, 29). Women are also more likely to receive symptom-based treatment over rhythm-control and less often referred for catheter ablation (30, 31). Addressing these sex-specific pharmacologic and access barriers in future research could enhance equity in multimorbidity management.

### 4.3 Race, ethnicity, and treatment access

Non-Hispanic White individuals with coexisting COPD and AF had the highest absolute mortality risk, consistent with the prior study in which AF was analyzed as the underlying cause of death (13). However, our broader multiple-cause approach also showed faster proportional increases among non-Hispanic Black and non-Hispanic American Indian or Alaska Native individuals, patterns that were less fully assessed in the prior study because of small-count suppression in non-White groups. These rising trends may reflect unequal access to diagnosis and treatment. Delayed DOAC initiation among Black and socioeconomically disadvantaged Medicare beneficiaries may partly explain disparities in AF outcomes (32, 33). Even as national data is well characterized, a lack of data disaggregation in Hispanic, Asian, as well as Indigenous groups is a concern. It is imperative that data becomes more nuanced in order to form culture-specific strategies accordingly. Various studies postulate that language differences, lack of confidence in the medical system, and the influence of drug-prescribing differences, act as contributing factors in disparities of anticoagulation in underserved populations (34, 35). Additionally, existing research highlights that air pollution disproportionately affects disadvantaged racial or ethnic groups, suggesting the interplay among environment and social inequity needs explicit acknowledgement (36).

### 4.4 Rurality, region and environmental factors

Singh et al. reported that most AF-related deaths with comorbid COPD occurred in metropolitan areas by count, but they also noted higher age-adjusted or per-population mortality in non-metropolitan areas in several strata (13). Our findings extend that observation by showing a consistent urban-rural gradient among older adults with coexisting AF and COPD. Non-metropolitan areas had both the highest AAMR and the fastest average annual increase. This distinction between counts and rates is important, as metropolitan areas may contribute more deaths because of larger population size, while non-metropolitan areas may carry higher per-population risk because of structural barriers to cardiopulmonary care. Limited access to clinicians in rural areas, particularly cardiologists and respiratory physicians for disease management and exacerbation care, as well as primary care or family medicine physicians for early recognition, referral coordination, chronic disease follow-up, and vaccination, may contribute to elevated cardiovascular and respiratory mortality (37, 38). In patients with COPD, these gaps may also reduce opportunities for smoking cessation support, influenza and pneumococcal vaccination, and prevention of exacerbation-related hospitalization, all of which are central to reducing mortality (39). Rural-urban disparities in cardiovascular outcomes stem from structural factors like income, transportation, and hospital access (40). Recent evidence indicates rural AF care lags in anticoagulation and rhythm management (41). Environmental exposures, including wildfire smoke and PM2.5 that increase cardiopulmonary risk in older adults, may further amplify these disparities (42). These exposures are also clinically relevant to COPD, as they can precipitate exacerbations, worsen hypoxemia, and increase susceptibility to respiratory infection, thereby creating conditions in which concomitant AF may further worsen prognosis (43, 44). A study in the Pacific Northwest found high PM2.5 during wildfires caused 15.1 additional respiratory deaths and 38.4 all-cause deaths per county (45). Consistent with this regional vulnerability, AF mortality has been reported to peak in the West and Midwest (46). Thus, regional-level differences reflect behavioral, environmental, and systemic factors. These findings support regionally tailored strategies that address both care access and environmental risk in older adults with coexisting AF and COPD.

### 4.5 Underlying causes and place of death

In the present study, COPD was the most common underlying cause of death among decedents with both AF and COPD. From 1999 to 2017, COPD-related mortality increased steadily, which may reflect population aging, repeated respiratory insults, and improved recognition and recording of COPD on death certificates. The subsequent plateau from 2017 to 2020 may be related to wider use of guideline-recommended inhaled therapies, better prevention and treatment of exacerbations, and increasing stability in cause-of-death coding practices. Between 1999 and 2020, the number of deaths with COPD as the underlying cause reached 140,168, which was 3.8 times the number of deaths with AF as the underlying cause. These findings indicate that, in this population, AF more often functions as a contributing condition within advanced cardiopulmonary disease rather than as the primary cause of death. Among patients hospitalized for acute exacerbations of COPD, concomitant AF has been associated with higher in-hospital mortality, longer hospital stay, and greater healthcare resource use (47). Respiratory infections may further amplify this vulnerability. In a population-based COPD cohort, new-onset AF was associated with higher risks of subsequent pneumonia, pneumonia requiring mechanical ventilation, and all-cause mortality (48). In a nationwide study of community-acquired pneumonia, AF diagnosed during hospitalization remained associated with higher in-hospital mortality after propensity-score matching, whereas pre-existing AF was no longer associated with excess mortality after matching (49). The increase in mortality with AF and COPD is better understood as a rise in the overall mortality burden associated with older age, multimorbidity, and infection- or exacerbation-triggered clinical deterioration. Within this setting, AF may represent both a marker of physiologic vulnerability and a potential contributor to hemodynamic worsening during respiratory decompensation.

Place-of-death patterns changed substantially over time. Deaths at home rose from 16.6% in 1999 to 34.6% in 2020 and became slightly more common than deaths in medical facilities by 2020. In contrast, the share of deaths in nursing or long-term care declined despite an increase in absolute counts, suggesting that the observed shift was driven mainly by growth in home-based death rather than a proportional movement toward institutional long-term care. This trend towards dying at home and in hospices is consistent with overall national data and continues during the pandemic era (50). In the management of COPD and AF patients, the earlier integration of goals-of-care talks and palliative care management is a major move towards optimizing care. Future studies could focus on crafting a new framework in managing COPD-AF that is a uniquely challenging condition in advance care planning, particularly in relation to risk of sudden death due to concomitant AF.

### 4.6 Rural and home-based care

The non-metropolitan excess and the shift toward home deaths suggest that a growing share of mortality occurs outside settings where specialist cardiopulmonary care is readily available. For older adults with co-listed AF and COPD, rural residence may delay treatment adjustment until symptoms have already worsened. Beyond expanding telehealth access, policy efforts should support a shared cardiopulmonary review pathway within rural primary care. Tele-cardiology could guide anticoagulation oversight and rhythm management, while tele-pulmonology could support post-exacerbation COPD optimization and assessment for rehabilitation or home respiratory support (16, 39, 51). In non-metropolitan areas, where travel distance and limited specialist access make repeated hospital-centered care difficult, this model could link tele-specialty support with primary-care-led community follow-up. A cluster-randomized trial in rural China showed that a telemedicine-based, village doctor-led integrated care model reduced composite cardiovascular events compared with usual care (52). These reviews may be most useful after hospitalization, emergency department treatment, or outpatient exacerbation, when separate AF and COPD visits may miss the interaction between respiratory instability and cardiac vulnerability. The rise in home deaths also supports earlier integration of palliative care. For patients with advanced cardiopulmonary disease or repeated deterioration, palliative care should be framed as symptom support and planning rather than withdrawal of disease-directed treatment. Earlier involvement allows clinicians to address goals of care and home-based crisis planning while patients can still participate in decisions (53). The observed shift toward home deaths therefore highlights the need for stronger community-based cardiopulmonary support in this high-risk population.

### 4.7 Forecast and therapeutic implications

The forecasts should be interpreted in conjunction with the validation results. Point forecast performance was generally good across the overall, sex-stratified, and age-stratified models, as reflected by low RMSE, MAE, MAPE, and sMAPE values during 2015–2020. Negative mean error values indicated a mild tendency toward underprediction, suggesting that future mortality may be slightly underestimated. Prediction interval coverage also varied by subgroup, indicating nonuniform interval calibration. Given the small number of validation observations, these forecasts are more informative for the direction and relative magnitude of future mortality trends than for precise single-year estimates.

Our projections suggest a continued rise in mortality rates with coexisting AF and COPD through 2030. COPD-directed management should therefore be emphasized in this high-risk group. In older adults with COPD, guideline-based inhaler therapy with long-acting bronchodilators, including combinations of long-acting beta-agonists and long-acting muscarinic antagonists (LABA/LAMA), can reduce symptoms and exacerbation risk, while inhaled corticosteroid (ICS)-containing regimens are considered for patients with recurrent exacerbations and appropriate inflammatory profiles (39). This is particularly relevant because acute exacerbations and respiratory infections may destabilize patients with coexisting AF and COPD and contribute to excess mortality. Large randomized trials have shown that single-inhaler triple therapy with LABA/LAMA/ICS reduces moderate or severe COPD exacerbations compared with dual therapy in patients with symptomatic COPD and exacerbation risk (54, 55). At the same time, AF-directed management remains essential. The AF “ABC” management strategy, where A stands for anticoagulation, B for symptom management, and C for comorbidity management, has been shown to reduce morbidity and mortality in patients with AF (56). Elderly individuals with multimorbidity treated with the ABC strategy via mobile telecommunication tools had fewer stroke and cardiovascular events (57). Real-world data from a multicenter European registry also support ABC adherence as an independent predictor of lower mortality and fewer hospitalizations, including in patients with multimorbidity (58). DOACs may offer advantages over warfarin in many older adults because of their favorable safety profile and lower risk of intracranial bleeding (59). Cardioselective beta-blockers are generally considered effective and safe when rate control is needed in patients with AF and coexisting COPD (60). However, rate or rhythm management alone is unlikely to be sufficient if underlying COPD remains poorly controlled. Optimization of inhaled maintenance therapy, prevention of exacerbations, and careful follow-up after respiratory events are also likely to influence prognosis in this population. Smoking trends should also be considered when interpreting future mortality patterns, because tobacco exposure remains a major driver of COPD progression and cardiopulmonary risk. Although cigarette smoking has declined substantially in the United States, the older COPD population still reflects decades of cumulative tobacco exposure, and disparities in cessation and tobacco-related disease may continue to shape future mortality (61). Smoking cessation remains a central nonpharmacologic intervention to slow lung-function decline and reduce COPD-related risk. GOLD-based management in older adults with AF and COPD therefore requires integrated care that includes smoking cessation, vaccination, inhaler optimization, and prevention of exacerbations in addition to AF-directed treatments (17). Telehealth and remote monitoring platforms have been shown to improve adherence, symptom detection, and risk factor control in both AF and COPD care (62, 63), indicating that a combined tele-respiratory and cardiac model may be beneficial. Treatment strategies should therefore be individualized to balance thromboembolic risk, bleeding risk, COPD severity, exacerbation burden, inhaler adherence, and polypharmacy concerns in frail older patients.

### 4.8 Interpreting the rising mortality signal

Death certificates cannot capture the full clinical course behind co-listed AF and COPD. The rising mortality signal likely reflects several secular processes acting together rather than a single causal pathway. Population aging remains central, since AF prevalence rises steeply with age and COPD burden remains concentrated in older adults with cumulative tobacco exposure (2, 5). The growing prevalence of obesity, diabetes, frailty, and multimorbidity may also increase the number of patients vulnerable to shared cardiopulmonary complications (14). Improved survival after acute coronary syndromes and heart failure may leave more patients living long enough to develop AF and COPD as coexisting chronic conditions (64, 65). Treatment patterns may also contribute. Β_2_-agonist exposure can increase heart rate and sympathetic tone, and methylxanthines such as theophylline may increase arrhythmic susceptibility in selected patients, although the present data cannot directly assess medication use, dose, or indication (7).

These population-level explanations are biologically plausible in COPD-AF overlap. Lung hyperinflation, pulmonary hypertension, and right-sided pressure loading can increase atrial stretch, creating a structural substrate for AF initiation and persistence. Systemic inflammation, oxidative stress, hypoxemia, and autonomic imbalance can further promote atrial remodeling and electrical instability (7). Once AF develops, loss of atrial contraction and irregular ventricular response may reduce cardiopulmonary reserve, worsen exertional dyspnea, and impair tolerance of COPD exacerbations or pneumonia (47, 48). This bidirectional interaction may help explain why co-listed AF and COPD identify a clinically fragile subgroup rather than two unrelated conditions recorded on the same death certificate.

### 4.9 Death-certificate coding and ascertainment bias

Our findings should be interpreted within the constraints of death-certificate surveillance. Multiple-cause-of-death data are well suited for population monitoring, but they do not adjudicate whether AF or COPD directly caused death in any individual case. AF ascertainment is sensitive to data source. In linked routine-care data, combining primary and secondary care records identified about one third more AF cases than hospital data alone, which shows how readily AF can be missed when surveillance depends on a single recording stream (66). Coding position also matters. In U.S. mortality analyses, AF or atrial flutter coded as an underlying cause has shown a different temporal pattern from AF or atrial flutter coded as a contributing cause (19). COPD is also incompletely captured on death certificates. In a population-based spirometry-defined COPD cohort, COPD was recorded on only 28.2% of death certificates overall and on 57.1% even among severe disease (67). In the TORCH trial, death-certificate primary or secondary cause agreed with adjudicated cause in 80% of informative deaths, which still leaves meaningful room for misclassification (68). Coding practice likely changed over time as diagnostic intensity increased, including broader use of natriuretic peptide testing and cardiac imaging to identify heart failure, atrial enlargement, pulmonary hypertension, and other cardiopulmonary abnormalities that may make AF, COPD, and related conditions more likely to be documented on death certificates. COVID-19 guidance also introduced U07.1 and explicit instructions for reporting pre-existing contributing conditions in 2020. For these reasons, the present study should be interpreted as mortality with co-listed AF and COPD, not mortality due to comorbid AF and COPD.

### 4.10 Limitations

Firstly, the data on deaths obtained from CDC WONDER is potentially prone to misreporting or underreporting, especially deaths that involve more than one contributing cause. Secondly, ICD-10 codes are not exhaustive in terms of capturing deaths involving COPD and AF. Furthermore, there are certain variables that cannot be measured in this analysis that could potentially influence the observed pattern of mortality. Additionally, the ARIMA model used for forecasting mortality trends is based on historical data and does not account for external shocks, such as the COVID-19 pandemic or changes in healthcare policies. These factors may significantly disrupt the trends, and their exclusion from the model limits the accuracy of long-term projections. Future studies may consider incorporating such variables to improve model robustness and the validity of mortality forecasts.

## 5 Conclusion

Among older adults in the U.S., mortality involving coexisting COPD and AF has increased over the past two decades, with persistent heterogeneity by sex, race, geography, and urbanicity. These trends reflect the interplay of biological, environmental, and health-system factors. Integrating tele-health, remote monitoring, and comorbidity-aware care pathways could improve patient outcomes by addressing these disparities.

## Data Availability

The datasets presented in this study can be found in online repositories. The names of the repository/repositories and accession number(s) can be found below: All data used in this study are publicly available from the CDC WONDER database (https://wonder.cdc.gov).
