# Supplementary material for: Mortality trends and disparities in older U.S. adults with atrial fibrillation and COPD: a 1999–2020 CDC WONDER analysis with forecast to 2030
Source: Front Cardiovasc Med. 2026 Jun 23;13:1811188. doi: 10.3389/fcvm.2026.1811188 (PMC13337513; doi:10.3389/fcvm.2026.1811188)
Supplement: Supplementary file 1 [file Datasheet1.docx]

**Supplementary Table 1.** Deaths and crude mortality rates stratified by sex among older adults with coexisting AF and COPD in the United States, 1999–2020

| Year | Overall, deaths (CMR) | Female, deaths (CMR) | Male, deaths (CMR) | Population |
| --- | --- | --- | --- | --- |
| 1999 | 9425 (27.09) | 4484 (21.88) | 4941 (34.55) | 34797841 |
| 2000 | 9920 (28.35) | 4764 (23.15) | 5156 (35.78) | 34991753 |
| 2001 | 10433 (29.56) | 5132 (24.81) | 5301 (36.29) | 35290291 |
| 2002 | 11215 (31.57) | 5477 (26.39) | 5738 (38.87) | 35522207 |
| 2003 | 11448 (31.92) | 5648 (27.04) | 5800 (38.73) | 35863529 |
| 2004 | 11764 (32.49) | 5759 (27.4) | 6005 (39.54) | 36203319 |
| 2005 | 13142 (35.86) | 6542 (30.86) | 6600 (42.72) | 36649798 |
| 2006 | 13342 (35.9) | 6722 (31.37) | 6620 (42.08) | 37164107 |
| 2007 | 13985 (36.97) | 6920 (31.84) | 7065 (43.91) | 37825711 |
| 2008 | 15302 (39.46) | 7642 (34.43) | 7660 (46.19) | 38777621 |
| 2009 | 15381 (38.82) | 7745 (34.27) | 7636 (44.85) | 39623175 |
| 2010 | 16651 (41.35) | 8259 (36.06) | 8392 (48.33) | 40267984 |
| 2011 | 18047 (43.6) | 9029 (38.5) | 9018 (50.26) | 41394141 |
| 2012 | 19246 (44.61) | 9447 (38.83) | 9799 (52.08) | 43145356 |
| 2013 | 20994 (46.96) | 10243 (40.8) | 10751 (54.85) | 44704074 |
| 2014 | 21219 (45.89) | 10409 (40.2) | 10810 (53.12) | 46243211 |
| 2015 | 23573 (49.36) | 11730 (43.98) | 11843 (56.15) | 47760852 |
| 2016 | 24470 (49.69) | 11832 (43.1) | 12638 (57.99) | 49244195 |
| 2017 | 27031 (53.15) | 13060 (46.16) | 13971 (61.92) | 50858679 |
| 2018 | 28381 (54.13) | 13760 (47.25) | 14621 (62.73) | 52431193 |
| 2019 | 30321 (56.09) | 14405 (48.04) | 15916 (66.11) | 54058263 |
| 2020 | 34123 (61.31) | 16021 (51.95) | 18102 (72.93) | 55659365 |
| Total | 399413 (43.02) | 195030 (36.96) | 204383 (50.99) | 928476665 |

Abbreviations: AF, atrial fibrillation; COPD, chronic obstructive pulmonary disease; CMR, crude mortality rate per 100,000

**Supplementary Table 2.** Deaths and crude mortality rates stratified by ten-year age groups among older adults with coexisting AF and COPD in the United States, 1999–2020

| **Year** | **65-74 Years**, **deaths (CMR)** | **75-84 Years**, **deaths (CMR)** | **≥85 Years**, **deaths (CMR)** |
| --- | --- | --- | --- |
| 1999 | 1917 (10.41) | 4351 (35.59) | 3157 (76) |
| 2000 | 1966 (10.69) | 4606 (37.26) | 3348 (78.97) |
| 2001 | 1969 (10.71) | 4844 (38.46) | 3620 (83.94) |
| 2002 | 2042 (11.11) | 5251 (41.14) | 3922 (89.77) |
| 2003 | 2111 (11.41) | 5256 (40.76) | 4081 (91.38) |
| 2004 | 2132 (11.42) | 5442 (41.89) | 4190 (92.17) |
| 2005 | 2290 (12.13) | 5994 (45.84) | 4858 (103.51) |
| 2006 | 2261 (11.77) | 6161 (47.05) | 4920 (101.11) |
| 2007 | 2445 (12.41) | 6254 (47.79) | 5286 (104.89) |
| 2008 | 2726 (13.29) | 6573 (50.27) | 6003 (115.54) |
| 2009 | 2816 (13.26) | 6483 (49.78) | 6082 (113.32) |
| 2010 | 3052 (14.06) | 6861 (52.53) | 6738 (122.66) |
| 2011 | 3348 (14.89) | 7263 (55.13) | 7436 (129.61) |
| 2012 | 3659 (15.26) | 7643 (57.59) | 7944 (134.93) |
| 2013 | 4067 (16.13) | 8284 (61.61) | 8643 (143.08) |
| 2014 | 4300 (16.29) | 8215 (60.04) | 8704 (141.25) |
| 2015 | 4853 (17.62) | 8950 (64.28) | 9770 (155.4) |
| 2016 | 5230 (18.27) | 9358 (65.75) | 9882 (154.88) |
| 2017 | 5868 (19.77) | 10323 (70.19) | 10840 (167.58) |
| 2018 | 6064 (19.89) | 10889 (70.73) | 11428 (174.62) |
| 2019 | 6634 (21.07) | 11678 (73.13) | 12009 (181.82) |
| 2020 | 7592 (23.33) | 13400 (81.45) | 13131 (197.21) |
| Total | 79342 (15.54) | 164079 (54.97) | 155992 (130.52) |

Abbreviations: AF, atrial fibrillation; COPD, chronic obstructive pulmonary disease; CMR, crude mortality rate per 100,000

**Supplementary Table 3.** Deaths and crude mortality rates stratified by race and ethnicity among older adults with coexisting AF and COPD in the United States, 1999–2020

| Year | non-Hispanic White, deaths (CMR) | non-Hispanic Black or African American, deaths (CMR) | non-Hispanic Asian or Pacific Islander, deaths (CMR) | non-Hispanic American Indian or Alaska Native, deaths (CMR) | Hispanic or Latino, deaths (CMR) |
| --- | --- | --- | --- | --- | --- |
| 1999 | 9018 (29.12) | 328 (11.45) | 64 (7.77) | 15 (NA) | 169 (10.19) |
| 2000 | 9479 (30.51) | 349 (12.07) | 70 (8.06) | 22 (13.78) | 144 (8.31) |
| 2001 | 9993 (31.98) | 340 (11.53) | 81 (8.69) | 19 (NA) | 155 (8.42) |
| 2002 | 10768 (34.32) | 345 (11.55) | 74 (7.5) | 28 (16.2) | 200 (10.38) |
| 2003 | 10947 (34.64) | 379 (12.48) | 93 (8.92) | 29 (16.06) | 187 (9.26) |
| 2004 | 11260 (35.37) | 376 (12.19) | 91 (8.28) | 37 (19.61) | 216 (10.23) |
| 2005 | 12547 (39.04) | 449 (14.25) | 111 (9.53) | 35 (17.64) | 237 (10.7) |
| 2006 | 12726 (39.14) | 451 (14.03) | 125 (10.18) | 40 (19.24) | 271 (11.7) |
| 2007 | 13346 (40.41) | 472 (14.37) | 132 (10.19) | 35 (16.01) | 246 (10.15) |
| 2008 | 14593 (43.17) | 532 (15.76) | 137 (10.04) | 40 (17.29) | 303 (11.9) |
| 2009 | 14648 (42.49) | 515 (14.86) | 164 (11.39) | 54 (22.07) | 307 (11.47) |
| 2010 | 15812 (45.21) | 601 (16.97) | 186 (12.4) | 52 (20.38) | 380 (13.66) |
| 2011 | 17160 (47.9) | 614 (16.68) | 204 (12.66) | 69 (25.09) | 412 (13.88) |
| 2012 | 18324 (49.16) | 697 (18.13) | 160 (9.25) | 65 (22.21) | 451 (14.35) |
| 2013 | 19887 (51.65) | 779 (19.35) | 252 (13.54) | 76 (24.35) | 538 (16.15) |
| 2014 | 20087 (50.63) | 816 (19.33) | 217 (10.79) | 99 (29.75) | 517 (14.56) |
| 2015 | 22326 (54.7) | 869 (19.64) | 268 (12.38) | 110 (31.03) | 646 (17.12) |
| 2016 | 23107 (55.06) | 939 (20.3) | 311 (13.67) | 113 (30.06) | 674 (16.98) |
| 2017 | 25478 (58.99) | 1106 (22.87) | 307 (12.6) | 140 (35.14) | 803 (19.1) |
| 2018 | 26705 (60.12) | 1167 (23.18) | 362 (14.16) | 147 (34.82) | 824 (18.65) |
| 2019 | 28447 (62.3) | 1327 (25.24) | 383 (14.23) | 164 (36.74) | 903 (19.47) |
| 2020 | 31803 (67.85) | 1720 (31.38) | 445 (15.71) | 155 (32.78) | 1161 (23.78) |
| Total | 378461 (47.12) | 15171 (18.22) | 4237 (11.8) | 1544 (25.51) | 9744 (14.96) |

Abbreviations: AF, atrial fibrillation; COPD, chronic obstructive pulmonary disease; CMR, crude mortality rate per 100,000

NA = Not available

**Supplementary Table 4.** Deaths and crude mortality rates stratified by census regions among older adults with coexisting AF and COPD in the United States, 1999–2020

| Year | Northeast, deaths (CMR) | Midwest, deaths (CMR) | South, deaths (CMR) | West, deaths (CMR) |
| --- | --- | --- | --- | --- |
| 1999 | 2159 (29.39) | 2290 (27.77) | 2982 (24.13) | 1994 (29.11) |
| 2000 | 2189 (29.69) | 2471 (29.92) | 3234 (26) | 2026 (29.27) |
| 2001 | 2331 (31.59) | 2588 (31.25) | 3396 (26.99) | 2118 (30.06) |
| 2002 | 2439 (33.03) | 2761 (33.3) | 3677 (28.95) | 2338 (32.72) |
| 2003 | 2358 (31.89) | 2865 (34.37) | 3844 (29.88) | 2381 (32.76) |
| 2004 | 2483 (33.56) | 2961 (35.35) | 3685 (28.25) | 2635 (35.68) |
| 2005 | 2651 (35.8) | 3332 (39.56) | 4334 (32.63) | 2825 (37.48) |
| 2006 | 2605 (35.09) | 3376 (39.69) | 4376 (32.29) | 2985 (38.86) |
| 2007 | 2681 (35.77) | 3547 (41.13) | 4633 (33.47) | 3124 (39.73) |
| 2008 | 3003 (39.37) | 3840 (43.69) | 5049 (35.43) | 3410 (42.05) |
| 2009 | 2946 (38.12) | 3938 (44.15) | 5227 (35.76) | 3270 (39.12) |
| 2010 | 3218 (41.23) | 4064 (45.04) | 5622 (37.75) | 3747 (43.84) |
| 2011 | 3300 (41.56) | 4542 (49.4) | 6071 (39.48) | 4134 (46.54) |
| 2012 | 3637 (44.25) | 4702 (49.42) | 6731 (41.83) | 4176 (44.8) |
| 2013 | 3722 (44.07) | 5087 (51.95) | 7395 (44.21) | 4790 (49.18) |
| 2014 | 3802 (43.98) | 5195 (51.64) | 7354 (42.34) | 4868 (47.87) |
| 2015 | 4143 (46.82) | 5677 (55) | 8316 (46.21) | 5437 (51.32) |
| 2016 | 4007 (44.27) | 5748 (54.3) | 8996 (48.31) | 5719 (52.06) |
| 2017 | 4294 (45.83) | 6426 (58.8) | 9990 (51.96) | 6321 (55.77) |
| 2018 | 4399 (45.98) | 6813 (60.62) | 10707 (53.87) | 6462 (54.99) |
| 2019 | 4544 (46.29) | 7133 (61.74) | 11834 (57.65) | 6810 (56) |
| 2020 | 5322 (52.96) | 8035 (67.78) | 13305 (62.74) | 7461 (59.46) |
| Total | 72233 (40.19) | 97391 (47.02) | 140758 (40.98) | 89031 (44.92) |

Abbreviations: AF, atrial fibrillation; COPD, chronic obstructive pulmonary disease; CMR, crude mortality rate per 100,000

**Supplementary Table 5.** Deaths and crude mortality rates stratified by urban-rural classification among older adults with coexisting AF and COPD in the United States, 1999–2020

| **Year** | **Large metropolitan, deaths (CMR)** | **Small-medium metropolitan, deaths (CMR)** | **Non-metropolitan, deaths (CMR)** |
| --- | --- | --- | --- |
| 1999 | 4414 (25.38) | 3021 (28.12) | 1990 (29.88) |
| 2000 | 4492 (25.67) | 3285 (30.38) | 2143 (32.08) |
| 2001 | 4745 (26.87) | 3425 (31.34) | 2263 (33.75) |
| 2002 | 5086 (28.62) | 3650 (33.12) | 2479 (36.84) |
| 2003 | 5124 (28.55) | 3815 (34.24) | 2509 (37.03) |
| 2004 | 5353 (29.57) | 3861 (34.24) | 2550 (37.36) |
| 2005 | 5867 (32.02) | 4480 (39.15) | 2795 (40.62) |
| 2006 | 5909 (31.9) | 4602 (39.46) | 2831 (40.57) |
| 2007 | 6157 (32.65) | 4835 (40.66) | 2993 (42.29) |
| 2008 | 6745 (34.85) | 5299 (43.38) | 3258 (45.2) |
| 2009 | 6740 (34.02) | 5340 (42.75) | 3301 (45.09) |
| 2010 | 7407 (36.75) | 5790 (45.58) | 3454 (46.61) |
| 2011 | 8019 (38.58) | 6265 (47.97) | 3763 (49.86) |
| 2012 | 8377 (38.55) | 6655 (48.86) | 4214 (54.06) |
| 2013 | 9233 (40.88) | 7282 (51.57) | 4479 (56.03) |
| 2014 | 9371 (39.99) | 7206 (49.27) | 4642 (56.7) |
| 2015 | 10284 (42.38) | 8145 (53.86) | 5144 (61.44) |
| 2016 | 10574 (42.19) | 8653 (55.38) | 5243 (61.29) |
| 2017 | 11455 (44.04) | 9778 (60.73) | 5798 (66.31) |
| 2018 | 11829 (44.09) | 10167 (61.1) | 6385 (71.25) |
| 2019 | 12565 (45.35) | 10832 (63.08) | 6924 (75.44) |
| 2020 | 14387 (50.37) | 12046 (68.03) | 7690 (81.89) |
| Total | 174133 (37.18) | 138432 (47.39) | 86848 (51.7) |

Abbreviations: AF, atrial fibrillation; COPD, chronic obstructive pulmonary disease; CMR, crude mortality rate per 100,000

**Supplementary Table 6.** Absolute number of deaths stratified by place of death among older adults with coexisting AF and COPD in the United States, 1999–2020

| **Year** | **Medical facility** | **Decedent’s home** | **Hospice facility** | **Nursing/Long-term care** | **Other** |
| --- | --- | --- | --- | --- | --- |
| 1999 | 5067 | 1560 | 0 | 2600 | 198 |
| 2000 | 5289 | 1755 | 0 | 2647 | 226 |
| 2001 | 5509 | 1843 | 0 | 2817 | 264 |
| 2002 | 5819 | 2027 | 0 | 2988 | 379 |
| 2003 | 5738 | 2198 | 15 | 3089 | 408 |
| 2004 | 5849 | 2330 | 42 | 3075 | 468 |
| 2005 | 6349 | 2667 | 148 | 3498 | 480 |
| 2006 | 6381 | 2739 | 221 | 3531 | 470 |
| 2007 | 6528 | 2949 | 351 | 3666 | 491 |
| 2008 | 7130 | 3185 | 429 | 3945 | 613 |
| 2009 | 6744 | 3382 | 514 | 3904 | 837 |
| 2010 | 7115 | 3877 | 702 | 4290 | 658 |
| 2011 | 7663 | 4312 | 841 | 4509 | 714 |
| 2012 | 7803 | 4809 | 1006 | 4863 | 761 |
| 2013 | 8185 | 5391 | 1189 | 5256 | 973 |
| 2014 | 8105 | 5768 | 1300 | 5288 | 758 |
| 2015 | 8838 | 6415 | 1646 | 5895 | 771 |
| 2016 | 8737 | 6976 | 1885 | 6042 | 828 |
| 2017 | 9609 | 7678 | 2088 | 6663 | 988 |
| 2018 | 9831 | 8436 | 2235 | 6867 | 1010 |
| 2019 | 10137 | 9524 | 2525 | 7019 | 1113 |
| 2020 | 11460 | 11808 | 2501 | 7014 | 1337 |
| Total | 163886 (41.03%) | 101629 (25.44%) | 19638 (4.92%) | 99466 (24.90%) | 14794 (3.70%) |

Abbreviations: AF, atrial fibrillation; COPD, chronic obstructive pulmonary disease

**Supplementary Table 7.** Age-adjusted mortality rates per 100,000 individuals stratified by sex among older adults with coexisting AF and COPD in the United States, 1999–2020

| Year | Overall | Female | Male |
| --- | --- | --- | --- |
| 1999 | 27.39 | 20.64 | 39.34 |
| 2000 | 28.52 | 21.73 | 40.56 |
| 2001 | 29.55 | 23.06 | 40.64 |
| 2002 | 31.4 | 24.46 | 43.45 |
| 2003 | 31.65 | 24.98 | 43.04 |
| 2004 | 32.14 | 25.28 | 43.74 |
| 2005 | 35.27 | 28.31 | 47.1 |
| 2006 | 35.25 | 28.71 | 45.99 |
| 2007 | 36.31 | 29.12 | 47.91 |
| 2008 | 38.97 | 31.6 | 50.64 |
| 2009 | 38.52 | 31.61 | 49.3 |
| 2010 | 41.05 | 33.29 | 53.19 |
| 2011 | 43.24 | 35.57 | 55.05 |
| 2012 | 44.98 | 36.47 | 57.9 |
| 2013 | 47.83 | 38.72 | 61.47 |
| 2014 | 47.13 | 38.68 | 59.77 |
| 2015 | 51.08 | 42.63 | 63.36 |
| 2016 | 51.88 | 42.17 | 65.81 |
| 2017 | 55.82 | 45.56 | 70.34 |
| 2018 | 56.91 | 46.79 | 71.23 |
| 2019 | 59.27 | 47.85 | 75.01 |
| 2020 | 65.29 | 52.36 | 83.08 |
| Total | 43.63 | 34.96 | 57.01 |

Abbreviations: AF, atrial fibrillation; COPD, chronic obstructive pulmonary disease

**Supplementary Table 8.** Age-adjusted mortality rates per 100,000 individuals stratified by race and ethnicity among older adults with coexisting AF and COPD in the United States, 1999–2020

| Year | non-Hispanic White | non-Hispanic Black or African American | non-Hispanic Asian or Pacific Islander | non-Hispanic American Indian or Alaska Native | Hispanic or Latino |
| --- | --- | --- | --- | --- | --- |
| 1999 | 29.17 | 12.14 | 10 | 13.61 | 12.62 |
| 2000 | 30.35 | 12.75 | 9.98 | 14.24 | 10.13 |
| 2001 | 31.54 | 12.14 | 10.5 | 14.84 | 10.37 |
| 2002 | 33.69 | 12.41 | 9.19 | 19.42 | 12.84 |
| 2003 | 33.84 | 13.5 | 11.28 | 19.23 | 11.3 |
| 2004 | 34.5 | 13.11 | 10.22 | 23.11 | 12.21 |
| 2005 | 37.85 | 15.38 | 11.42 | 20.26 | 12.73 |
| 2006 | 37.82 | 14.96 | 12.14 | 23.58 | 13.72 |
| 2007 | 39.06 | 15.55 | 11.76 | 19.82 | 11.93 |
| 2008 | 42.01 | 17 | 11.85 | 20.76 | 13.73 |
| 2009 | 41.52 | 15.77 | 13.54 | 27.53 | 13.13 |
| 2010 | 44.22 | 18.16 | 14.55 | 25.41 | 15.82 |
| 2011 | 46.82 | 18 | 14.32 | 30.83 | 15.74 |
| 2012 | 48.89 | 19.47 | 10.48 | 26.53 | 16.19 |
| 2013 | 51.92 | 20.95 | 15.41 | 27.59 | 18.11 |
| 2014 | 51.35 | 21.17 | 12.21 | 36.52 | 16.24 |
| 2015 | 55.89 | 21.46 | 13.98 | 34.6 | 18.95 |
| 2016 | 56.83 | 22.47 | 15.28 | 34.81 | 18.89 |
| 2017 | 61.21 | 25.43 | 14.07 | 43.63 | 21.29 |
| 2018 | 62.45 | 25.73 | 15.51 | 40.42 | 20.57 |
| 2019 | 65.08 | 27.93 | 15.71 | 43.14 | 21.7 |
| 2020 | 71.47 | 35.1 | 17.2 | 38.27 | 26.86 |
| Total | 47.17 | 19.74 | 13.59 | 30.12 | 17.15 |

Abbreviations: AF, atrial fibrillation; COPD, chronic obstructive pulmonary disease

Note: Values in red were not directly available but were estimated as the midpoint of the upper and lower limits of the 95% confidence interval, which was obtainable

**Supplementary Table 9.** Age-adjusted mortality rates per 100,000 individuals stratified by census regions among older adults with coexisting AF and COPD in the United States, 1999–2020

| Year | Northeast | Midwest | South | West |
| --- | --- | --- | --- | --- |
| 1999 | 29.12 | 27.49 | 24.9 | 29.82 |
| 2000 | 29.16 | 29.42 | 26.72 | 29.76 |
| 2001 | 30.72 | 30.53 | 27.71 | 30.31 |
| 2002 | 31.83 | 32.33 | 29.66 | 32.89 |
| 2003 | 30.39 | 33.22 | 30.61 | 32.84 |
| 2004 | 31.85 | 34.04 | 28.92 | 35.71 |
| 2005 | 33.72 | 37.95 | 33.34 | 37.3 |
| 2006 | 32.75 | 37.93 | 33.04 | 38.57 |
| 2007 | 33.43 | 39.3 | 34.22 | 39.37 |
| 2008 | 36.81 | 41.99 | 36.4 | 41.95 |
| 2009 | 35.81 | 42.53 | 36.9 | 39.14 |
| 2010 | 38.78 | 43.55 | 39.01 | 43.87 |
| 2011 | 38.96 | 47.82 | 40.71 | 46.54 |
| 2012 | 42.36 | 48.38 | 43.71 | 45.8 |
| 2013 | 42.51 | 51.45 | 46.64 | 50.81 |
| 2014 | 42.78 | 51.79 | 44.84 | 49.93 |
| 2015 | 46.21 | 55.16 | 49.29 | 53.97 |
| 2016 | 44.12 | 55.35 | 51.74 | 55.13 |
| 2017 | 45.88 | 60.73 | 55.71 | 59.51 |
| 2018 | 46.02 | 62.88 | 57.85 | 58.6 |
| 2019 | 46.76 | 64.62 | 61.99 | 60.17 |
| 2020 | 54.38 | 71.67 | 67.6 | 64.28 |
| Total | 38.96 | 46.56 | 42.87 | 46.22 |

Abbreviations: AF, atrial fibrillation; COPD, chronic obstructive pulmonary disease

**Supplementary Table 10.** Age-adjusted mortality rates per 100,000 individuals stratified by urban-rural classification among older adults with coexisting AF and COPD in the United States, 1999–2020

| **Year** | **Large metropolitan** | **Small-medium metropolitan** | **Non-metropolitan** |
| --- | --- | --- | --- |
| 1999 | 25.69 | 28.62 | 29.81 |
| 2000 | 25.82 | 30.69 | 31.89 |
| 2001 | 26.87 | 31.52 | 33.44 |
| 2002 | 28.40 | 33.11 | 36.51 |
| 2003 | 28.19 | 34.11 | 36.71 |
| 2004 | 29.13 | 34.00 | 37.09 |
| 2005 | 31.36 | 38.75 | 40.28 |
| 2006 | 31.08 | 38.89 | 40.36 |
| 2007 | 31.78 | 40.13 | 42.15 |
| 2008 | 34.12 | 42.98 | 45.32 |
| 2009 | 33.44 | 42.54 | 45.46 |
| 2010 | 36.15 | 45.30 | 47.14 |
| 2011 | 37.98 | 47.55 | 50.38 |
| 2012 | 38.59 | 49.31 | 55.32 |
| 2013 | 41.34 | 52.74 | 57.65 |
| 2014 | 40.80 | 50.73 | 58.87 |
| 2015 | 43.65 | 55.84 | 64.04 |
| 2016 | 43.80 | 57.96 | 64.50 |
| 2017 | 45.97 | 63.92 | 69.96 |
| 2018 | 46.13 | 64.40 | 75.26 |
| 2019 | 47.82 | 66.78 | 79.79 |
| 2020 | 53.54 | 72.62 | 87.21 |
| Total | 37.54 | 48.26 | 52.72 |

Abbreviations: AF, atrial fibrillation; COPD, chronic obstructive pulmonary disease

**Supplementary Table 11.** Age-adjusted mortality rates per 100,000 individuals stratified by states of the United States among older adults with coexisting AF and COPD, 1999–2020

| **State** | **AAMR per 100,000** | **Rank** | **Percentile** |
| --- | --- | --- | --- |
| Vermont | 77.57 | 1 | 100 |
| West Virginia | 70.61 | 2 | 98 |
| Rhode Island | 66.33 | 3 | 96 |
| Wyoming | 64.69 | 4 | 94 |
| Oklahoma | 64.22 | 5 | 92 |
| Oregon | 63.29 | 6 | 90 |
| Kentucky | 61.57 | 7 | 88 |
| Washington | 61.47 | 8 | 86 |
| Colorado | 60.71 | 9 | 84 |
| Ohio | 59.87 | 10 | 82 |
| Nebraska | 59.8 | 11 | 80 |
| Minnesota | 59.22 | 12 | 78 |
| New Hampshire | 57.59 | 13 | 76 |
| Tennessee | 54.88 | 14 | 74 |
| Maine | 54.75 | 15 | 72 |
| Indiana | 54.57 | 16 | 70 |
| North Dakota | 52.97 | 17 | 68 |
| Montana | 52.36 | 18 | 66 |
| Idaho | 51.71 | 19 | 64 |
| South Dakota | 51.53 | 20 | 62 |
| South Carolina | 51.04 | 21 | 60 |
| North Carolina | 50.52 | 22 | 58 |
| Texas | 48.61 | 23 | 56 |
| Maryland | 48.36 | 24 | 54 |
| Iowa | 48.33 | 25 | 52 |
| Alaska | 47.28 | 26 | 50 |
| Pennsylvania | 46.76 | 27 | 48 |
| California | 45.77 | 28 | 46 |
| Wisconsin | 43.8 | 29 | 44 |
| Mississippi | 43.64 | 30 | 42 |
| Delaware | 43.45 | 31 | 40 |
| Connecticut | 39.95 | 32 | 38 |
| Michigan | 39.36 | 33 | 36 |
| Missouri | 39.3 | 34 | 34 |
| New Jersey | 38.34 | 35 | 32 |
| Arkansas | 38.17 | 36 | 30 |
| Virginia | 38.03 | 37 | 28 |
| Alabama | 37.22 | 38 | 26 |
| Kansas | 35.05 | 39 | 24 |
| New Mexico | 35.04 | 39 | 24 |
| Illinois | 33.82 | 41 | 20 |
| Massachusetts | 32.89 | 42 | 18 |
| Florida | 31.43 | 43 | 16 |
| Arizona | 30.87 | 44 | 14 |
| New York | 29.8 | 45 | 12 |
| Nevada | 29.72 | 46 | 10 |
| Georgia | 29.06 | 47 | 8 |
| Louisiana | 28.73 | 48 | 6 |
| Utah | 23.18 | 49 | 4 |
| Hawaii | 22.75 | 50 | 2 |
| District of Columbia | 20.12 | 51 | 0 |

Abbreviations: AF, atrial fibrillation; COPD, chronic obstructive pulmonary disease; AAMR = Age-adjusted mortality rate

**Supplementary Table 12.** Age-adjusted mortality rates per 100,000 individuals stratified by states of the United States among older adults with coexisting AF and COPD, from1999 to 2020

| **States** | **1999 AAMR** | **2020 AAMR** | **% AAMR Change** |
| --- | --- | --- | --- |
| Oklahoma | 14.74 | 126.66 | 759.29 |
| South Dakota | 17 | 101.46 | 496.82 |
| Louisiana | 12.92 | 71.33 | 452.09 |
| Nebraska | 17.53 | 94.66 | 439.99 |
| Arkansas | 14.81 | 71.1 | 380.08 |
| Indiana | 23.84 | 103.59 | 334.52 |
| Mississippi | 20.89 | 89.24 | 327.19 |
| Arizona | 12.41 | 52.02 | 319.18 |
| Minnesota | 27.37 | 105.82 | 286.63 |
| Wisconsin | 23.48 | 88.82 | 278.28 |
| South Carolina | 25.33 | 87.9 | 247.02 |
| Wyoming | 45.6 | 155.42 | 240.83 |
| Iowa | 24.99 | 84.14 | 236.69 |
| Kentucky | 29.83 | 99.54 | 233.69 |
| Florida | 17.2 | 56.87 | 230.64 |
| Colorado | 35.36 | 111.96 | 216.63 |
| Oregon | 33.72 | 102.27 | 203.29 |
| Montana | 30.7 | 87.31 | 184.40 |
| Tennessee | 29.47 | 82.51 | 179.98 |
| Texas | 26.48 | 73.06 | 175.91 |
| Kansas | 23.54 | 63.42 | 169.41 |
| North Dakota | 26.77 | 68.78 | 156.93 |
| Missouri | 23.81 | 60.3 | 153.25 |
| Idaho | 31.29 | 76.71 | 145.16 |
| Washington | 32.86 | 78.01 | 137.40 |
| New York | 20.84 | 47.52 | 128.02 |
| New Mexico | 20.9 | 46.2 | 121.05 |
| Georgia | 17.93 | 38.58 | 115.17 |
| Illinois | 22.92 | 47 | 105.06 |
| New Jersey | 24.46 | 48.47 | 98.16 |
| Vermont | 46.84 | 92.34 | 97.14 |
| Michigan | 28.12 | 54.87 | 95.13 |
| Virginia | 28.88 | 55.88 | 93.49 |
| New Hampshire | 36.65 | 69.64 | 90.01 |
| Rhode Island | 48.36 | 91.84 | 89.91 |
| Massachusetts | 26.11 | 48.04 | 83.99 |
| District of Columbia | 16.47 | 29.97 | 81.97 |
| Nevada | 25.31 | 44.66 | 76.45 |
| West Virginia | 45.26 | 79.83 | 76.38 |
| California | 32.19 | 56.76 | 76.33 |
| Alabama | 29.04 | 49.73 | 71.25 |
| Utah | 23.04 | 38.83 | 68.53 |
| Pennsylvania | 38.6 | 64.83 | 67.95 |
| North Carolina | 36.44 | 60.69 | 66.55 |
| Ohio | 41.75 | 68.61 | 64.34 |
| Maryland | 46.02 | 75.48 | 64.02 |
| Maine | 46.35 | 74.01 | 59.68 |
| Delaware | 34.22 | 54.08 | 58.04 |
| Connecticut | 31.4 | 44.13 | 40.54 |
| Hawaii | 20.6 | 27.23 | 32.18 |
| Alaska | 53.47 | 40.04 | -25.12 |

Abbreviations: AF, atrial fibrillation; COPD, chronic obstructive pulmonary disease; AAMR, age-adjusted mortality rate

Note: Values in red were not directly available but were estimated as the midpoint of the upper and lower limits of the 95% confidence interval, which was obtainable

**Supplementary Table 13.** Age-adjusted mortality rates per 100,000 individuals stratified by underlying causes among older adults with coexisting AF and COPD, from1999 to 2020

| Year | Chronic obstructive pulmonary disease | Ischemic heart disease | Cancer | Atrial fibrillation | Heart failure | Stroke | Pulmonary heart disease | Chronic kidney disease |
| --- | --- | --- | --- | --- | --- | --- | --- | --- |
| 1999 | 9.94 | 6.19 | 2.58 | 1.89 | 1.05 | 0.89 | 0.16 | 0.06 |
| 2000 | 10.19 | 6.05 | 2.84 | 1.86 | 1.09 | 0.96 | 0.21 | 0.06 |
| 2001 | 10.31 | 6.32 | 2.9 | 2.22 | 1.21 | 0.95 | 0.16 | 0.06 |
| 2002 | 11.11 | 6.47 | 3.1 | 2.32 | 1.15 | 1.03 | 0.2 | 0.07 |
| 2003 | 11.45 | 6.23 | 3.11 | 2.24 | 1.25 | 0.88 | 0.23 | 0.07 |
| 2004 | 11.28 | 6.22 | 3.42 | 2.39 | 1.41 | 0.85 | 0.26 | 0.09 |
| 2005 | 12.46 | 6.84 | 3.5 | 2.61 | 1.48 | 0.96 | 0.26 | 0.07 |
| 2006 | 12.61 | 6.4 | 3.69 | 2.56 | 1.54 | 0.91 | 0.27 | 0.1 |
| 2007 | 12.94 | 6.31 | 3.97 | 3.63 | 0.58 | 0.72 | 0.27 | 0.13 |
| 2008 | 14.8 | 6.74 | 3.82 | 3.72 | 0.67 | 0.96 | 0.26 | 0.11 |
| 2009 | 14.64 | 6.28 | 4.12 | 3.69 | 0.63 | 0.88 | 0.29 | 0.09 |
| 2010 | 15.21 | 6.74 | 4.27 | 3.8 | 0.7 | 1.02 | 0.33 | 0.16 |
| 2011 | 16.19 | 6.74 | 4.53 | 4.19 | 0.77 | 1.06 | 0.36 | 0.2 |
| 2012 | 16.31 | 7.31 | 4.78 | 4.5 | 0.79 | 0.97 | 0.41 | 0.24 |
| 2013 | 17.63 | 7.54 | 4.77 | 4.69 | 0.85 | 1.03 | 0.44 | 0.25 |
| 2014 | 16.62 | 7.58 | 5.03 | 4.98 | 0.79 | 0.99 | 0.47 | 0.21 |
| 2015 | 18.1 | 7.83 | 5.15 | 5.46 | 0.98 | 1.1 | 0.41 | 0.23 |
| 2016 | 17.85 | 7.81 | 5.66 | 5.72 | 1.04 | 1.19 | 0.51 | 0.23 |
| 2017 | 19.85 | 8.35 | 5.71 | 5.75 | 1.28 | 1.29 | 0.53 | 0.26 |
| 2018 | 19.52 | 8.69 | 5.94 | 5.57 | 1.45 | 1.22 | 0.61 | 0.3 |
| 2019 | 19.88 | 9 | 6.44 | 5.77 | 1.72 | 1.13 | 0.67 | 0.34 |
| 2020 | 19.32 | 9.05 | 6.94 | 5.3 | 1.93 | 1.3 | 0.74 | 0.35 |

Abbreviations: AF, atrial fibrillation; COPD, chronic obstructive pulmonary disease

**Supplementary Table 14.** Joinpoint model selection and selected joinpoints

| **Variable** | **Group** | **Observations** | **Maximum joinpoints allowed** | **Selected joinpoints** | **Selected joinpoint year(s)** | **Joinpoint 95% CI** | **Selected model WBIC** | **Model selection method** | **Grid Search setting** |
| --- | --- | --- | --- | --- | --- | --- | --- | --- | --- |
| Sex | Female | 22 | 4 | 0 | — | — | 1.5500052 | WBIC | 2, 2, 0 |
| Sex | Male | 22 | 4 | 2 | 2009, 2018 | 2001–2015; 2013–2018 | 2.1711570 | WBIC | 2, 2, 0 |
| Age group | 65–74 years | 22 | 4 | 2 | 2007, 2018 | 2003–2011; 2010–2018 | 1.0337201 | WBIC | 2, 2, 0 |
| Age group | 75–84 years | 22 | 4 | 1 | 2018 | 2013–2018 | 1.6798421 | WBIC | 2, 2, 0 |
| Age group | ≥85 years | 22 | 4 | 0 | — | — | 1.5924767 | WBIC | 2, 2, 0 |
| Race/ethnicity | White | 22 | 4 | 1 | 2018 | 2004–2018 | 2.3953048 | WBIC | 2, 2, 0 |
| Race/ethnicity | Hispanic or Latino | 22 | 4 | 1 | 2018 | 2005–2018 | 1.1247088 | WBIC | 2, 2, 0 |
| Race/ethnicity | American Indian or Alaska Native | 22 | 4 | 0 | — | — | -0.1872432 | WBIC | 2, 2, 0 |
| Race/ethnicity | Asian or Pacific Islander | 22 | 4 | 0 | — | — | 0.5658605 | WBIC | 2, 2, 0 |
| Race/ethnicity | Black or African American | 22 | 4 | 1 | 2018 | 2017–2018 | 0.6891136 | WBIC | 2, 2, 0 |
| Urbanization | Large metropolitan | 22 | 4 | 0 | — | — | 1.8864206 | WBIC | 2, 2, 0 |
| Urbanization | Non-metropolitan | 22 | 4 | 1 | 2016 | 2008–2018 | 1.2716648 | WBIC | 2, 2, 0 |
| Urbanization | Small-medium metropolitan | 22 | 4 | 0 | — | — | 1.7525323 | WBIC | 2, 2, 0 |
| Census region | Northeast | 22 | 4 | 0 | — | — | 1.7723602 | WBIC | 2, 2, 0 |
| Census region | Midwest | 22 | 4 | 0 | — | — | 1.1076395 | WBIC | 2, 2, 0 |
| Census region | South | 22 | 4 | 1 | 2014 | 2010–2018 | 1.8891290 | WBIC | 2, 2, 0 |
| Census region | West | 22 | 4 | 0 | — | — | 1.3316026 | WBIC | 2, 2, 0 |
| Cause of death | COPD | 22 | 4 | 1 | 2017 | 2009–2018 | 2.3715493 | WBIC | 2, 2, 0 |
| Cause of death | AF | 22 | 4 | 1 | 2016 | 2014–2018 | 2.0602474 | WBIC | 2, 2, 0 |
| Cause of death | CKD | 22 | 4 | 3 | 2009, 2012, 2015 | 2004–2010; 2010–2014; 2014–2018 | 0.5828798 | WBIC | 2, 2, 0 |
| Cause of death | Cancer | 22 | 4 | 1 | 2018 | 2016–2018 | 0.2906321 | WBIC | 2, 2, 0 |
| Cause of death | HF | 22 | 4 | 2 | 2006, 2009 | 2004–2007; 2007–2011 | 2.8535920 | WBIC | 2, 2, 0 |
| Cause of death | IHD | 22 | 4 | 1 | 2009 | 2007–2012 | 1.1976350 | WBIC | 2, 2, 0 |
| Cause of death | PHD | 22 | 4 | 0 | — | — | 0.1316519 | WBIC | 2, 2, 0 |
| Cause of death | Stroke | 22 | 4 | 1 | 2007 | 2003–2011 | 1.3053904 | WBIC | 2, 2, 0 |
| State | Hawaii | 22 | 4 | 0 | — | — | 0.5583331 | WBIC | 2, 2, 0 |
| State | Alaska | 13 | 2 | 0 | — | — | 1.0595378 | WBIC | 2, 2, 0 |
| State | Oklahoma | 22 | 4 | 0 | — | — | 1.7415271 | WBIC | 2, 2, 0 |
| State | South Dakota | 20 | 3 | 0 | — | — | 0.3908448 | WBIC | 2, 2, 0 |

**Supplementary Table 15.** Pairwise parallelism test results

| **Variable** | **Group** | **Reference** | **Parallelism test *P* value** | **Interpretation** |
| --- | --- | --- | --- | --- |
| Sex | Male | Female | 0.000222 | Rejected parallelism |
| Age group | 75–84 years | 65–74 years | 0.000889 | Rejected parallelism |
| Age group | ≥85 years | 65–74 years | 0.000444 | Rejected parallelism |
| Race/ethnicity | Hispanic or Latino | White | 0.609111 | Failed to reject parallelism |
| Race/ethnicity | American Indian or Alaska Native | White | 0.658 | Failed to reject parallelism |
| Race/ethnicity | Asian or Pacific Islander | White | 0.013556 | Rejected parallelism |
| Race/ethnicity | Black or African American | White | 0.033556 | Rejected parallelism |
| Urbanization | Non-metropolitan | Large metropolitan | 0.000222 | Rejected parallelism |
| Urbanization | Small-medium metropolitan | Large metropolitan | 0.000222 | Rejected parallelism |
| Census region | Midwest | Northeast | 0.000222 | Rejected parallelism |
| Census region | South | Northeast | 0.000222 | Rejected parallelism |
| Census region | West | Northeast | 0.000222 | Rejected parallelism |
| Cause of death | AF | COPD | 0.000222 | Rejected parallelism |
| Cause of death | CKD | COPD | 0.001556 | Rejected parallelism |
| Cause of death | Cancer | COPD | 0.018667 | Rejected parallelism |
| Cause of death | HF | COPD | 0.000222 | Rejected parallelism |
| Cause of death | IHD | COPD | 0.000222 | Rejected parallelism |
| Cause of death | PHD | COPD | 0.000667 | Rejected parallelism |
| Cause of death | Stroke | COPD | 0.000222 | Rejected parallelism |
| State | Alaska | Hawaii | 0.002 | Rejected parallelism |
| State | Oklahoma | Hawaii | 0.000222 | Rejected parallelism |
| State | South Dakota | Hawaii | 0.000222 | Rejected parallelism |

**Supplementary Table 16.** BIC and AIC values for different ARIMA (p,d,q) model combinations by overall, sex, and age group

Abbreviations: BIC, Bayesian information criterion; AIC, Akaike information criterion; ARIMA, autoregressive integrated moving average

Note: Values with the minimum BIC are highlighted in red

**Supplementary Table 17.** ARIMA-based predicted age-adjusted mortality rates (AAMRs) among older adults with coexisting AF and COPD in the United States, 2015–2030

| **Year** | **Actual AAMR** | **Forecast AAMR** | **AAMR lower 95% confidence interval** | **AAMR upper 95% confidence interval** |
| --- | --- | --- | --- | --- |
| 2015 | 51.08 | 48.70 | 46.96 | 50.43 |
| 2016 | 51.88 | 52.08 | 50.16 | 54.01 |
| 2017 | 55.82 | 53.24 | 51.37 | 55.11 |
| 2018 | 56.91 | 56.72 | 54.58 | 58.86 |
| 2019 | 59.27 | 59.43 | 57.34 | 61.51 |
| 2020 | 65.29 | 61.58 | 59.55 | 63.60 |
| 2021 |  | 65.14 | 62.65 | 67.64 |
| 2022 |  | 69.57 | 66.80 | 72.34 |
| 2023 |  | 72.61 | 68.83 | 76.39 |
| 2024 |  | 74.51 | 69.53 | 79.48 |
| 2025 |  | 78.44 | 72.57 | 84.32 |
| 2026 |  | 80.83 | 73.56 | 88.10 |
| 2027 |  | 83.72 | 75.16 | 92.27 |
| 2028 |  | 86.96 | 77.05 | 96.87 |
| 2029 |  | 89.52 | 78.07 | 100.97 |
| 2030 |  | 92.62 | 79.66 | 105.58 |

Abbreviations: ARIMA, Integrated Moving Average; AF, atrial fibrillation; COPD, chronic obstructive pulmonary disease

Note: Values in red indicate observed AAMRs during the validation period (2015–2020)

**Supplementary Table 18.** ARIMA-based predicted age-adjusted mortality rates (AAMRs) among older adults with coexisting AF and COPD, stratified by sex in the United States in the United States, 2015–2030

| **Sex** | **Year** | **Actual AAMR** | **Forecast AAMR** | **AAMR lower 95% confidence interval** | **AAMR upper 95% confidence interval** |
| --- | --- | --- | --- | --- | --- |
| **Female** | 2015 | 42.63 | 40.94 | 39.49 | 42.39 |
|  | 2016 | 42.17 | 43.38 | 41.78 | 44.98 |
|  | 2017 | 45.56 | 44.83 | 43.19 | 46.48 |
|  | 2018 | 46.79 | 46.80 | 45.17 | 48.42 |
|  | 2019 | 47.85 | 48.67 | 47.09 | 50.24 |
|  | 2020 | 52.36 | 50.19 | 48.61 | 51.77 |
|  | 2021 |  | 52.69 | 50.92 | 54.47 |
|  | 2022 |  | 54.88 | 53.02 | 56.74 |
|  | 2023 |  | 57.06 | 54.97 | 59.15 |
|  | 2024 |  | 59.25 | 56.76 | 61.74 |
|  | 2025 |  | 61.43 | 58.39 | 64.48 |
|  | 2026 |  | 63.62 | 59.89 | 67.35 |
|  | 2027 |  | 65.80 | 61.29 | 70.32 |
|  | 2028 |  | 67.99 | 62.60 | 73.38 |
|  | 2029 |  | 70.18 | 63.83 | 76.52 |
|  | 2030 |  | 72.36 | 65.00 | 79.73 |
| **Male** | 2015 | 63.36 | 63.16 | 60.77 | 65.56 |
|  | 2016 | 65.81 | 64.53 | 62.24 | 66.83 |
|  | 2017 | 70.34 | 65.63 | 63.32 | 67.95 |
|  | 2018 | 71.24 | 76.14 | 73.21 | 79.08 |
|  | 2019 | 75.01 | 74.19 | 70.96 | 77.43 |
|  | 2020 | 83.08 | 77.45 | 74.28 | 80.62 |
|  | 2021 |  | 83.86 | 79.99 | 87.73 |
|  | 2022 |  | 88.49 | 83.68 | 93.29 |
|  | 2023 |  | 93.81 | 87.64 | 99.99 |
|  | 2024 |  | 96.45 | 87.97 | 104.94 |
|  | 2025 |  | 100.99 | 90.67 | 111.31 |
|  | 2026 |  | 105.38 | 92.89 | 117.86 |
|  | 2027 |  | 108.86 | 93.88 | 123.84 |
|  | 2028 |  | 113.19 | 95.79 | 130.60 |
|  | 2029 |  | 117.30 | 97.25 | 137.34 |
|  | 2030 |  | 121.13 | 98.28 | 143.98 |

Abbreviations: ARIMA, Integrated Moving Average; AF, atrial fibrillation; COPD, chronic obstructive pulmonary disease

Note: Values in red indicate observed AAMRs during the validation period (2015–2020)

**Supplementary Table 19.** ARIMA-based predicted age-adjusted mortality rates (AAMRs) among older adults with coexisting AF and COPD, stratified by ten-year age groups in the United States in the United States, 2015–2030

| **Age** | **Year** | **Actual AAMR** | **Forecast AAMR** | **AAMR lower 95% confidence interval** | **AAMR upper 95% confidence interval** |
| --- | --- | --- | --- | --- | --- |
| **65-74 Years** | 2015 | 17.62 | 16.88 | 16.31 | 17.46 |
|  | 2016 | 18.27 | 18.04 | 17.38 | 18.70 |
|  | 2017 | 19.77 | 18.88 | 18.24 | 19.53 |
|  | 2018 | 19.89 | 20.68 | 19.93 | 21.42 |
|  | 2019 | 21.07 | 21.21 | 20.41 | 22.01 |
|  | 2020 | 23.33 | 21.72 | 20.94 | 22.51 |
|  | 2021 |  | 23.66 | 22.67 | 24.65 |
|  | 2022 |  | 25.44 | 24.13 | 26.76 |
|  | 2023 |  | 26.88 | 25.00 | 28.75 |
|  | 2024 |  | 27.74 | 25.08 | 30.39 |
|  | 2025 |  | 29.48 | 26.19 | 32.77 |
|  | 2026 |  | 30.61 | 26.47 | 34.74 |
|  | 2027 |  | 31.84 | 26.84 | 36.84 |
|  | 2028 |  | 33.36 | 27.49 | 39.24 |
|  | 2029 |  | 34.49 | 27.62 | 41.36 |
|  | 2030 |  | 35.87 | 28.00 | 43.74 |
| **75-84 Years** | 2015 | 64.28 | 61.67 | 58.43 | 64.91 |
|  | 2016 | 65.75 | 66.07 | 62.68 | 69.47 |
|  | 2017 | 70.19 | 67.52 | 64.24 | 70.80 |
|  | 2018 | 70.73 | 72.12 | 68.69 | 75.54 |
|  | 2019 | 73.13 | 72.58 | 69.20 | 75.97 |
|  | 2020 | 81.45 | 75.00 | 71.70 | 78.30 |
|  | 2021 |  | 84.38 | 80.06 | 88.69 |
|  | 2022 |  | 87.30 | 80.77 | 93.84 |
|  | 2023 |  | 90.23 | 81.69 | 98.77 |
|  | 2024 |  | 93.16 | 82.67 | 103.64 |
|  | 2025 |  | 96.08 | 83.65 | 108.51 |
|  | 2026 |  | 99.01 | 84.61 | 113.41 |
|  | 2027 |  | 101.93 | 85.53 | 118.34 |
|  | 2028 |  | 104.86 | 86.41 | 123.31 |
|  | 2029 |  | 107.79 | 87.24 | 128.33 |
|  | 2030 |  | 110.71 | 88.03 | 133.40 |
| **≥85 Years** | 2015 | 155.40 | 148.78 | 141.73 | 155.82 |
|  | 2016 | 154.88 | 157.47 | 150.05 | 164.90 |
|  | 2017 | 167.58 | 160.63 | 153.38 | 167.88 |
|  | 2018 | 174.62 | 173.49 | 165.80 | 181.18 |
|  | 2019 | 181.82 | 179.40 | 171.92 | 186.87 |
|  | 2020 | 197.21 | 192.81 | 185.44 | 200.19 |
|  | 2021 |  | 201.62 | 194.22 | 209.02 |
|  | 2022 |  | 214.55 | 205.63 | 223.47 |
|  | 2023 |  | 224.28 | 210.78 | 237.77 |
|  | 2024 |  | 231.97 | 213.58 | 250.35 |
|  | 2025 |  | 244.72 | 222.23 | 267.22 |
|  | 2026 |  | 252.26 | 223.46 | 281.07 |
|  | 2027 |  | 262.98 | 228.82 | 297.15 |
|  | 2028 |  | 273.34 | 232.94 | 313.73 |
|  | 2029 |  | 281.83 | 234.61 | 329.04 |
|  | 2030 |  | 293.06 | 239.32 | 346.80 |

Abbreviations: ARIMA, Integrated Moving Average; AF, atrial fibrillation; COPD, chronic obstructive pulmonary disease

Note: Values in red indicate observed AAMRs during the validation period (2015–2020)

**Supplementary Figure S1.** Trends in crude mortality rates among older adults with coexisting AF and COPD, stratified by ten-year age groups in the United States, 1999–2020


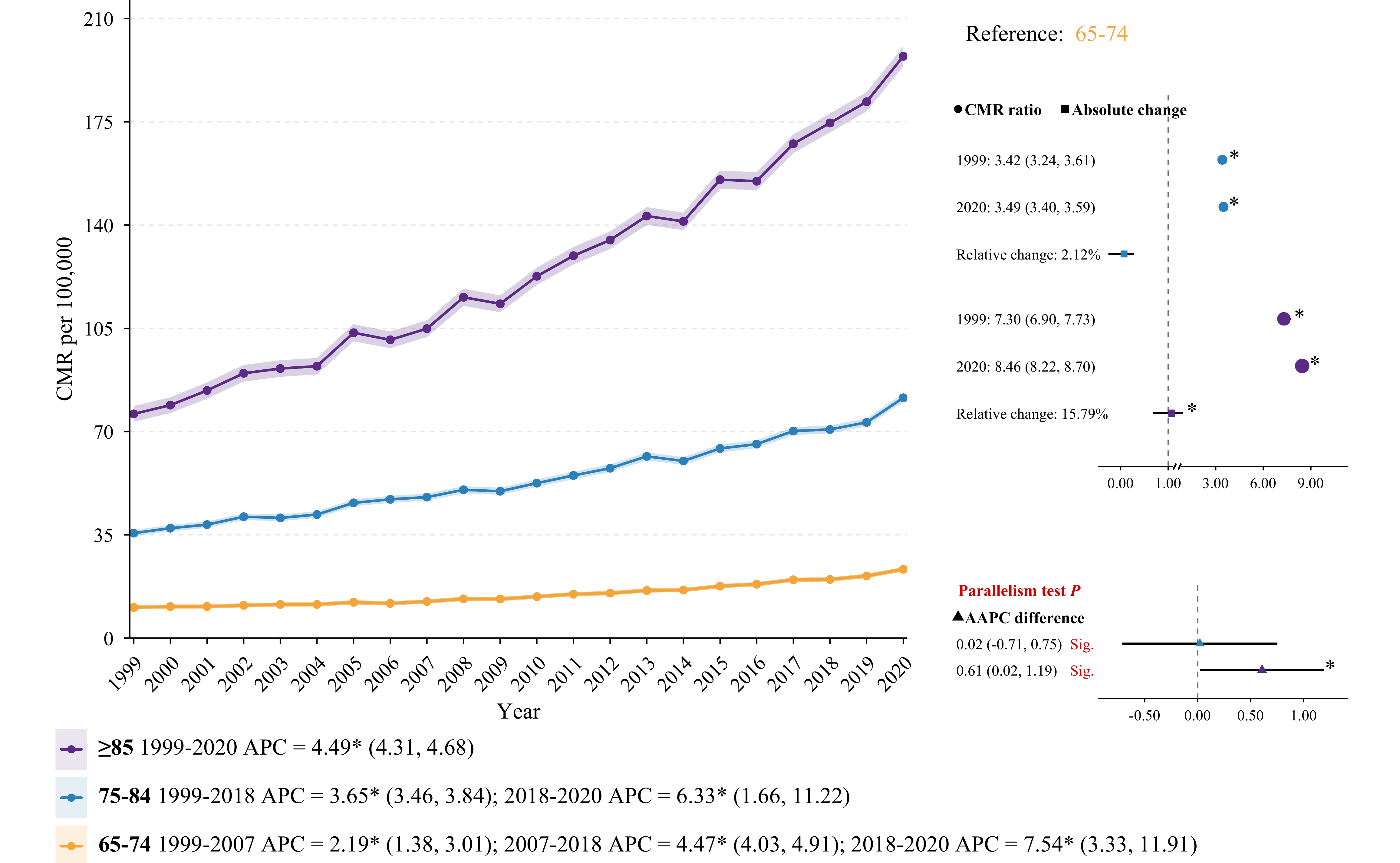
Abbreviations: AF, atrial fibrillation; COPD, chronic obstructive pulmonary disease

**Supplementary Figure S2.** Trends in age-adjusted mortality rates (AAMRs) among older adults with coexisting AF and COPD, stratified by urban–rural classification in the United States, 1999–2020


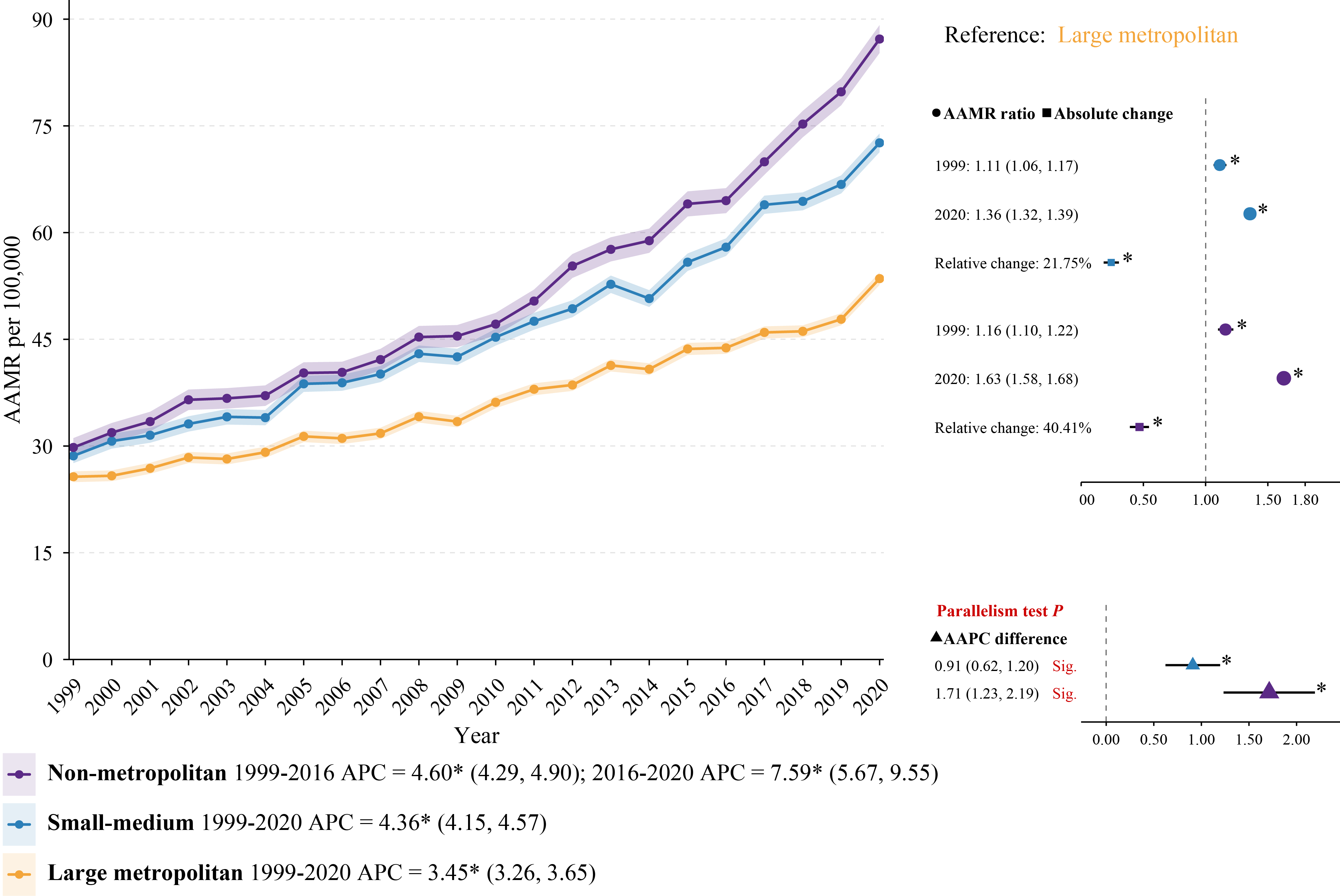
Abbreviations: AF, atrial fibrillation; COPD, chronic obstructive pulmonary disease

**Supplementary Figure S3.** Trends in deaths among older adults with coexisting AF and COPD, stratified by place of death in the United States, 1999–2020


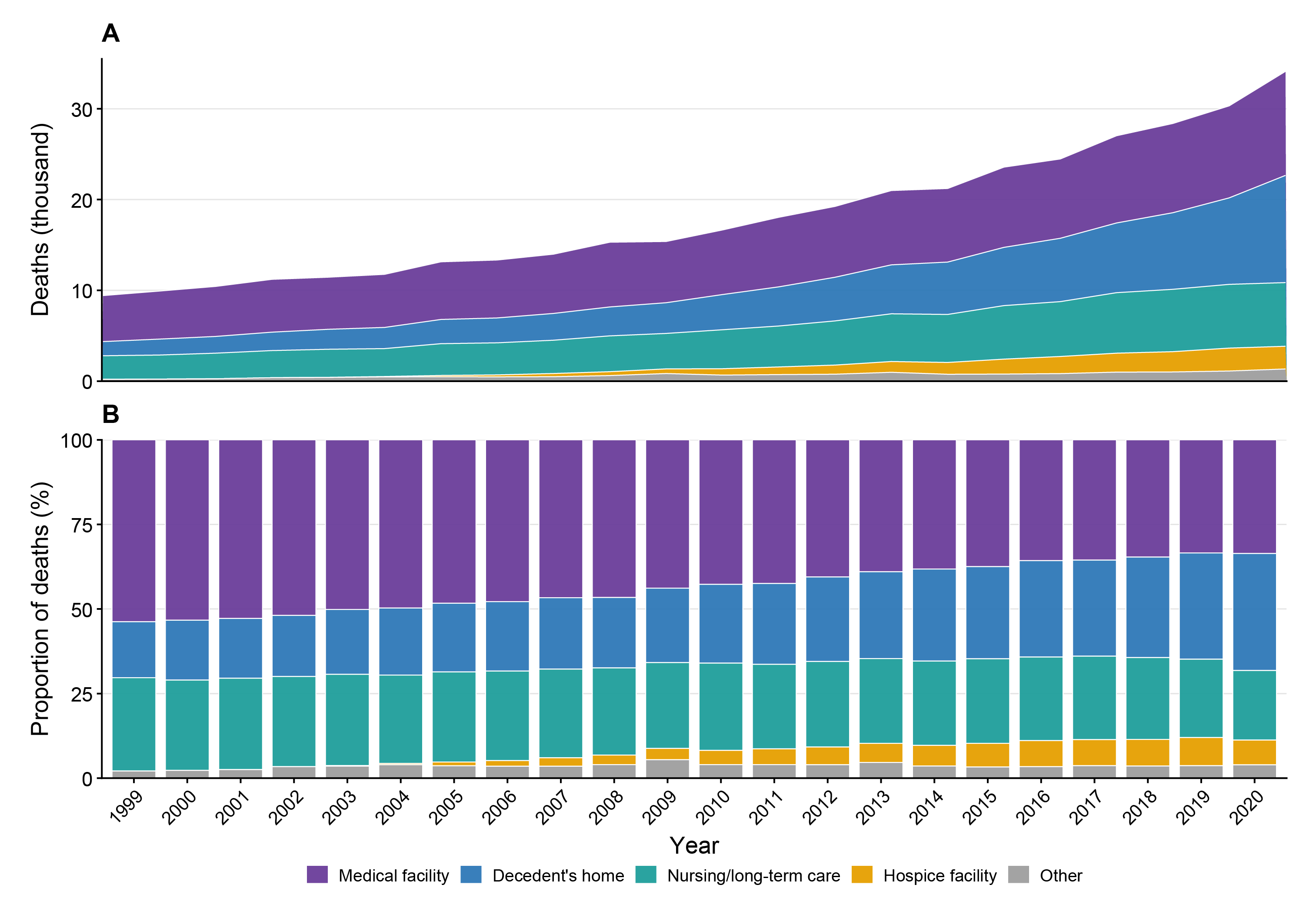
Abbreviations: AF, atrial fibrillation; COPD, chronic obstructive pulmonary disease

Note: Trends in deaths of hospice facility is 2003-2020

**Supplementary Figure S4.** Trends in age-adjusted mortality rates (AAMRs) among older adults with coexisting AF and COPD in the United States, for the states with the top two and bottom two % AAMR increases, 1999–2020


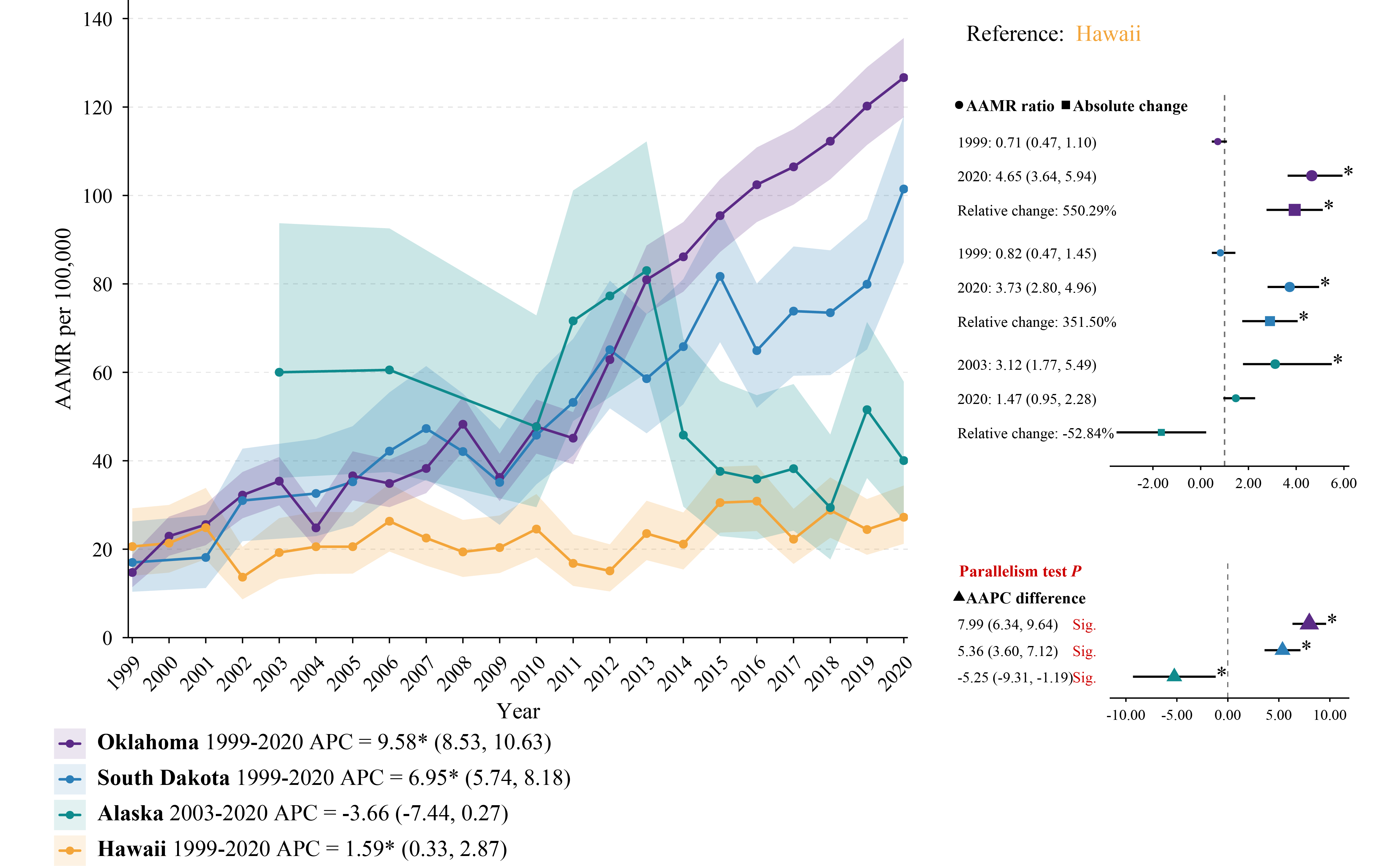
Abbreviations: AF, atrial fibrillation; COPD, chronic obstructive pulmonary disease

Note: The trend of Alaska was calculated for the period 2003–2020

**Supplementary Figure S5.** WBIC heatmap for candidate Joinpoint models

**
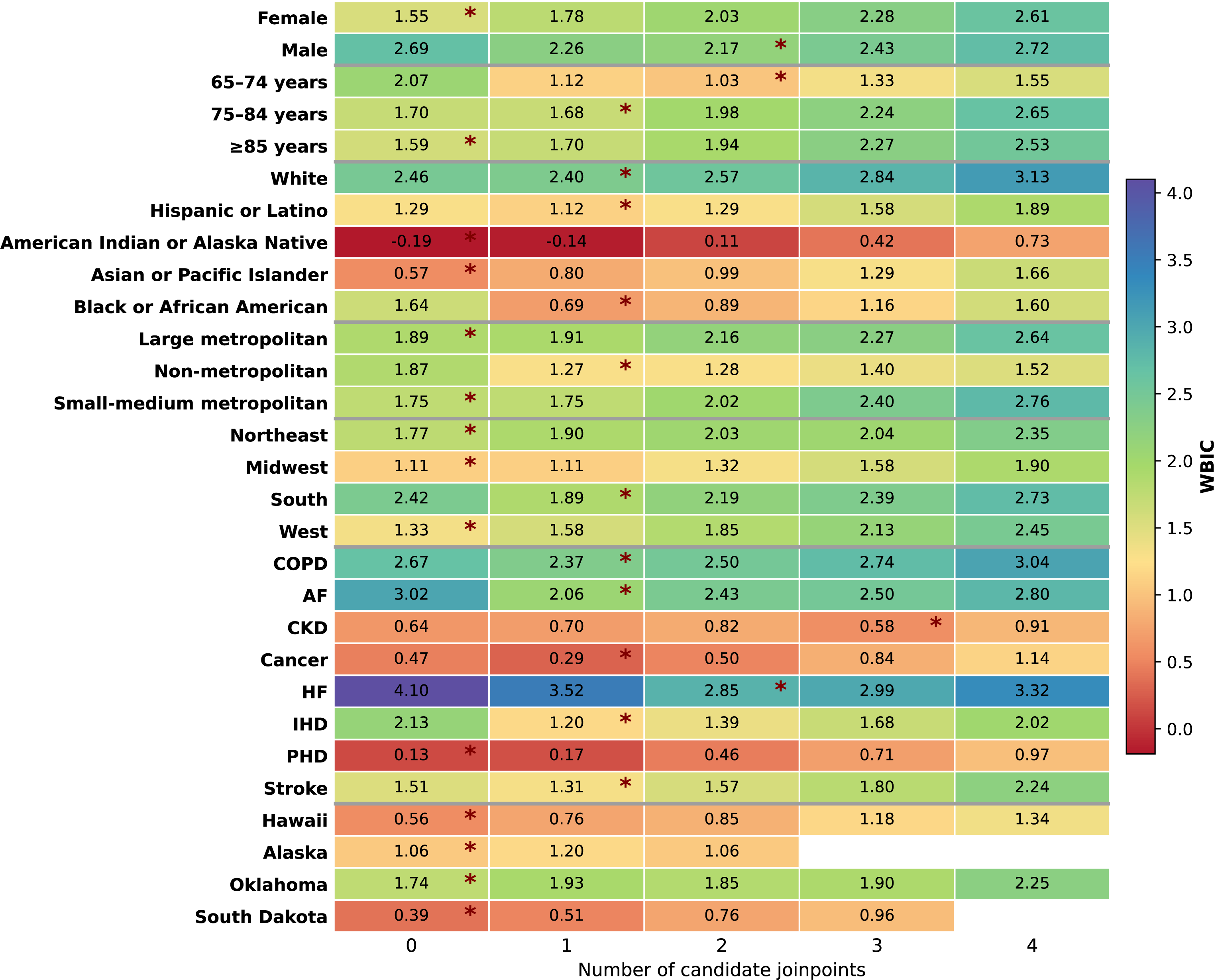
**Abbreviations: WBIC, weighted Bayesian information criterion

Note: Asterisks indicate the optimal number of joinpoints selected by WBIC
